# Upregulation of MLK4 promotes migratory and invasive potential of breast cancer cells

**DOI:** 10.1038/s41388-018-0618-0

**Published:** 2018-12-14

**Authors:** Anna A. Marusiak, Monika K. Prelowska, Dawid Mehlich, Michal Lazniewski, Klaudia Kaminska, Adam Gorczynski, Aleksandra Korwat, Olga Sokolowska, Hanna Kedzierska, Jakub Golab, Wojciech Biernat, Dariusz Plewczynski, John Brognard, Dominika Nowis

**Affiliations:** 1 Laboratory of Experimental Medicine, Centre of New Technologies, University of Warsaw, Warsaw, Poland; 2 Postgraduate School of Molecular Medicine, Medical University of Warsaw, Warsaw, Poland; 3 Genomic Medicine, Medical University of Warsaw, Warsaw, Poland; 4 Laboratory of Functional and Structural Genomics, Centre of New Technologies, University of Warsaw, Warsaw, Poland; 5 Department of Physical Chemistry, Faculty of Pharmacy, Medical University of Warsaw, Warsaw, Poland; 6 Department of Pathomorphology, Medical University of Gdansk, Gdańsk, Poland; 7 Department of Immunology, Medical University o``f Warsaw, Warsaw, Poland; 8 Centre for Preclinical Research and Technology, Medical University of Warsaw, Warsaw, Poland; 9 Faculty of Mathematics and Information Science, Warsaw University of Technology, Warsaw, Poland; 10 National Cancer Institute, Frederick, MD USA

**Keywords:** Mechanisms of disease, Breast cancer

## Abstract

Metastasis to distant organs is a major cause for solid cancer mortality, and the acquisition of migratory and invasive phenotype is a key factor in initiation of malignancy. In this study we investigated the contribution of Mixed-Lineage Kinase 4 (MLK4) to aggressive phenotype of breast cancer cells. Our TCGA cancer genomic data analysis revealed that amplification or mRNA upregulation of MLK4 occurred in 23% of invasive breast carcinoma cases. To find the association between MLK4 expression and the specific subtype of breast cancer, we performed a transcriptomic analysis of multiple datasets, which showed that MLK4 is highly expressed in triple-negative breast cancer compared to other molecular subtypes. Depletion of MLK4 in cell lines with high MLK4 expression impaired proliferation and anchorage-dependent colony formation. Moreover, silencing of MLK4 expression significantly reduced the migratory potential and invasiveness of breast cancer cells as well as the number of spheroids formed in 3D cultures. These in vitro findings translate into slower rate of tumor growth in mice upon MLK4 knock-down. Furthermore, we established that MLK4 activates NF-κB signaling and promotes a mesenchymal phenotype in breast cancer cells. Immunohistochemical staining of samples obtained from breast cancer patients revealed a strong positive correlation between high expression of MLK4 and metastatic potential of tumors, which was predominantly observed in TNBC subgroup. Taken together, our results show that high expression of MLK4 promotes migratory and invasive phenotype of breast cancer and may represent a novel target for anticancer treatment.

## Introduction

Breast cancer is a highly heterogeneous disease classified into several subgroups varying on molecular characterization, treatment responses and clinical outcomes [1]. The most commonly used system of classification, that usually determines treatment choice, is based on the expression of estrogen receptor (ER), progesterone receptor (PR) and human epidermal growth factor receptor 2 (HER2). Histopathological assessments of samples have their limitations and an additional approach, based on the patterns of gene expression, further aids in treatment stratification [2]. Gene expression studies have identified several molecular subtypes of breast cancer including luminal A, luminal B, HER2-enriched, basal-like and normal breast-like [1, 3]. The subgroup which is characterized by the absence of ER/PR and the lack of HER2 overexpression is called triple-negative breast cancer (TNBC). TNBC represents about 10-20% of all breast cancers, and approximately 80% of TNBCs are simultaneously classified as basal-like [4, 5]. TNBC is a heterogeneous subgroup linked with an aggressive phenotype, frequent metastasis and poor prognosis, where no targeted therapies have been developed so far [6–8].

MLK4 (*MAP3K21/KIAA1804*) is a serine/threonine kinase that belongs to Mixed-Lineage Kinase (MLK) family. MLK1-4 are activated by environmental stress, cytokines and growth factors to phosphorylate JNK, p38 and ERK signaling pathways [9, 10]. Despite growing knowledge about the role of MLKs in tumorigenesis, MLK4 is still the least studied member of that family. MLK4 has been shown to inhibit the activation of MAPK pathways (including p38, JNK, and ERK), negatively regulate MLK3 kinase activity, and act as a suppressor of cell invasion in ovarian cancer [11–13]. Contrary to these reports, it has been demonstrated that MLK4 can directly phosphorylate MEK leading to activation of the ERK pathway [14, 15]. Moreover, activation of the MEK/ERK pathway by MLK1-4 has implications in mediating the resistance to vemurafenib treatment in BRAF-V600E positive melanoma [10]. A recent study demonstrated that MLK4 regulates activation of a transcription factor NF-κB and determines mesenchymal phenotype of glioma stem cells associated with the aggressiveness of the disease [16]. These findings emphasize the tumor-promoting activity of MLK4; however, we recently reported that MLK4 can also suppress tumorigenesis in colon cancer [15]. Thus, the function of MLK4 in cancer progression might be more complex and dependent on many factors, including cell-type, the cancer-specific mutations profile, and the stage of cancer development. Here, we provide evidence demonstrating that MLK4 is highly expressed in breast cancer clinical samples, predominantly in TNBC. We found that depletion of MLK4 resulted in decreased proliferation and cell cycle arrest in TNBC cell lines. Moreover, we demonstrated that MLK4 knock-down inhibited the migratory and invasive properties of TNBC cells in vitro and attenuated tumor growth in vivo. Mechanistically, we showed that MLK4 activated NF-κB signaling and promoted the expression of EMT (epithelial–mesenchymal transition) markers in cancer cells. Finally, we observed that high expression of MLK4 was positively correlated with the occurrence of lymph node metastasis in TNBC patients, as revealed by the immunohistochemical analysis of breast cancer samples.

## Results

### MLK4 is upregulated in breast cancer, predominantly in TNBC

**Fig. 1:**
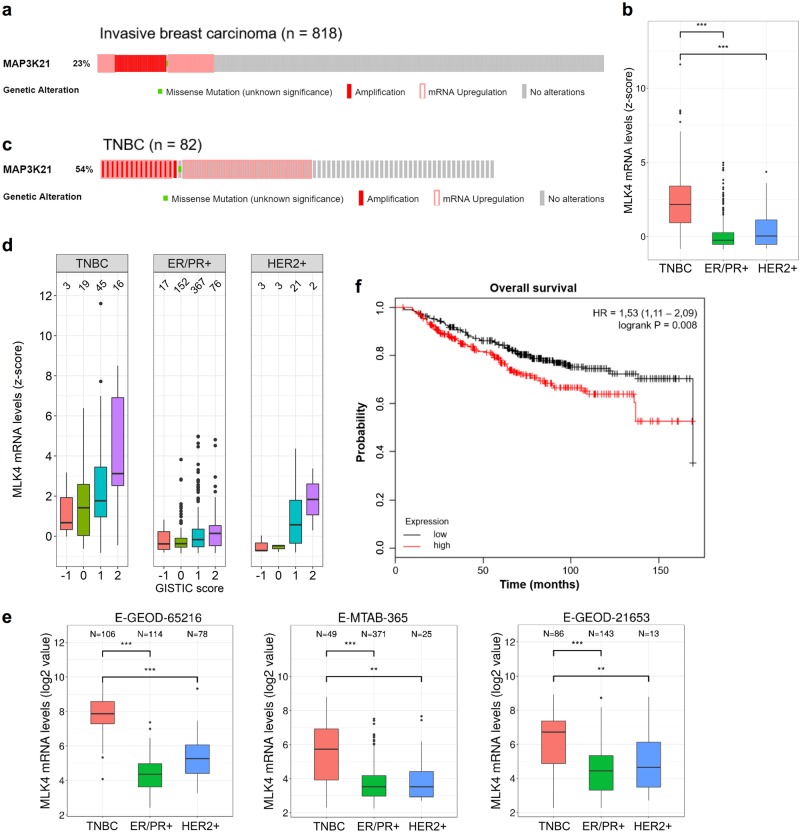
MLK4 is upregulated in invasive breast carcinoma. **a**, **c** Amplification and mRNA expression profiling of MLK4 in all invasive breast carcinoma cases (**a**) and TNBC cases (**c**) from TCGA dataset. Graphic illustrations taken from cBio Portal. **b** Expression of MLK4 in breast cancer subtypes for samples from TCGA. *P*-values between selected distributions were calculated using Mann–Whitney *U* test. ***P < 0.001. **d** The difference in MLK4 expression in breast cancer subtypes with respect to copy number alterations of MLK4 gene. Copy number variation is provided in form of a GISTIC score: −1 (deletion), 0 (diploid), 1 (low-level gain), 2 (high-level amplification). The number over given box represents a size of a population constituting a given distribution. **e** MLK4 expression in three datasets. Samples were assigned to specific subgroups of breast cancer based on the provided immunohistochemical status of ER, PR, and HER2 receptors. The obtained mRNA levels are shown with respect to different subgroups of breast cancer. *P*-values between selected distributions were calculated using Mann–Whitney *U* test. **P < 0.01, ***P < 0.001. **f** Probability of overall survival in breast cancer patients expressing high or low MLK4 levels assessed using KMplotter, with auto-selected best cutoff. Graphic illustrations taken from Kmplot.com

In order to evaluate the expression of MLK4 in breast cancer patients we analyzed 818 samples available in TCGA, which showed gene amplification and mRNA upregulation of MLK4 (*MAP3K21*) in invasive breast carcinoma at a frequency of 23%, in contrast to lower frequency of alterations (6–7%) in the rest of the MLK family members (Fig. 1a and Supplementary Fig. 1a) [17–19]. Next, we sought to determine a relationship between MLK4 mRNA levels and the specific subtypes of breast cancer. We found that MLK4 was highly upregulated in the samples of TNBC patients compared to other subtypes (Fig. 1b), and that MLK4 gene amplification and/or mRNA upregulation was present in more than 50% of TNBC samples in analyzed TCGA datasets (Fig. 1c). Additional analysis of TCGA tumor samples indicated that MLK4 mRNA overexpression was highly concordant with increased copy number for the *MAP3K21* gene, particularly in TNBC (Fig. 1d). Furthermore, the higher levels of MLK4 gene expression in TNBC compared to other breast cancer subtypes were confirmed by the analysis of three independent microarray datasets (Fig. 1e) [20–22]. The differential gene expression analysis between TNBC, HER2+ or ER/PR+ samples identified MLK4 among the top 1% of all genes with the highest probability of being differentially expressed between TNBC and other breast cancer subtypes (Supplementary Fig. 1b-d). To investigate the relationship between MLK4 expression and clinical outcome of breast cancer patients, we used the KM-plotter, containing information from multiple microarray datasets [23]. The analysis performed on a cohort of breast cancer patients revealed that high MLK4 expression was associated with significantly shorter overall (OS) and recurrence-free survival (RFS) (Fig. 1f and Supplementary Fig. 2a) [23]. Furthermore, the association between high MLK4 expression level and poor RFS was also significant when only TNBC patients cohort was considered (the analysis for OS was not performed due to low number of TNBC samples) (Supplementary Fig. 2b). Noteworthy, no association between the expression of the other members of MLK family and OS in breast cancer patients was observed (Supplementary Fig. 2c).

### MLK4 knock-down decreases clonogenic potential of breast cancer cells

**Fig. 2:**
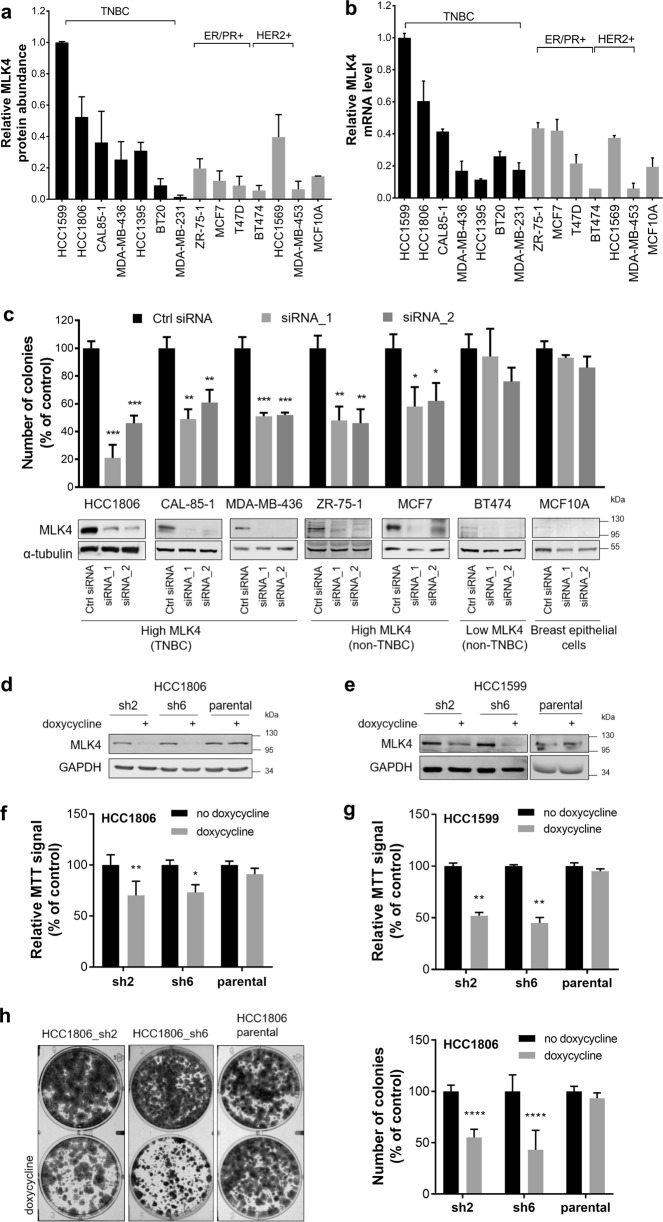
MLK4 knock-down decreases anchorage-dependent growth of breast cancer cells. **a**, **b** MLK4 protein abundance (**a**) and mRNA expression levels (**b**) in a panel of breast cancer cell lines. Densitometry analysis was performed from three independent experiments. α-tubulin was used to normalize the results (**a**). B2M and RPL29 were used as reference genes to determine relative mRNA expression levels (**b**). Error bars indicate ± SEM from three independent experiments. **c** A panel of breast cancer cell lines and MCF10A were transfected with siRNA against MLK4 or control siRNA. After 72 h cells were stained with crystal violet and results were quantified by absorbance (upper graph) or cells were lysed and analyzed by western blotting. Error bars indicate ± SEM from three independent experiments (*n* = 3). Statistical comparison of values was performed using the unpaired two-tailed *t*-test. ***P < 0.001, **P < 0.01, *P < 0.05. **d**, **e** HCC1806_sh2, HCC1806_sh6, parental HCC1806 (**d**), and HCC1599_sh2, HCC1599_sh6, parental HCC1599 (**e**) cells were treated with 1 μg/ml doxycycline for 72 h. Whole cell lysates were analyzed by western blotting. **f**, **g** HCC1806_sh2, HCC1806_sh6, parental HCC1806 (**f**), and HCC1599_sh2, HCC1599_sh6, parental HCC1599 (**g**) cells were treated with 1 μg/ml doxycycline for 6 days. Viability of cells was determined by MTT assay. Error bars indicate ± SEM from three independent experiments (*n* = 9). Statistical comparison of values was performed using the unpaired two-tailed *t*-test. **P < 0.01, *P < 0.05. **h** HCC1806_sh2, HCC1806_sh6 and parental HCC1806 cells were seeded at low density and were grown with or without 1 μg/ml doxycycline. After 2 weeks, cells were stained with crystal violet (pictures on the left), and quantified by absorbance. Error bars indicate ± SEM from five independent experiments (*n* = 5). Statistical comparison of values was performed using the unpaired two-tailed *t*-test. ****P < 0.0001

In order to find the most suitable cell line models for further work, that recapitulate high MLK4 expression observed in primary tumors, we assessed MLK4 levels in breast cancer cell lines by western blotting and RT-qPCR. We identified cell lines with high and low expression levels of MLK4 in all subtypes of breast cancer cell lines (Fig. 2a, b and Supplementary Fig. 3). We then assessed the functional role of MLK4 by knocking-down MLK4 in several cell lines including breast cancer cell lines with high MLK4 expression (HCC1806, CAL-85-1, MDA-MB-436, ZR-75-1, and MCF7), low MLK4 expression (BT474) and in immortalized breast epithelial cells (MCF10A). The silencing of MLK4 impaired anchorage-dependent colony formation assay of cells expressing high levels of MLK4, but not control cell lines, BT474 or MCF10A that have low MLK4 expression (Fig. 2c). Taking into the consideration our transcriptomic analysis showing MLK4 upregulation in TNBC patients (Fig. 1b, e) and current lack of targeted therapies for this group of patients, we decided to focus our study on TNBC.

We selected two TNBC cell lines with high endogenous expression of MLK4, HCC1806, and HCC1599, to generate cell lines with inducible knock-down of MLK4, where the expression of turboRFP and shRNA targeting *MAP3K21* (sh2 and sh6) is regulated by doxycycline (Fig. 2d, e). First, we performed MTT assays, which showed that silencing of MLK4 induced cytotoxic/cytostatic effects on both cell lines (Fig. 2f, g). Similarly, the ability to form colonies in a long-term anchorage-dependent assay was reduced after MLK4 depletion in the HCC1806 cell line (Fig. 2h). The long-term anchorage-dependent assay was not performed in HCC1599 as it is a suspension cell line and thus the number of additional phenotypic assays is limited. No cytotoxic/cytostatic effects or significant reduction in colony formation in the parental cell lines HCC1806 and HCC1599 treated with doxycycline was observed (Fig. 2f–h) suggesting that effects mentioned above are due to decreased levels of MLK4.

### MLK4 depletion leads to cell cycle arrest in MLK4-high TNBC cells

**Fig. 3:**
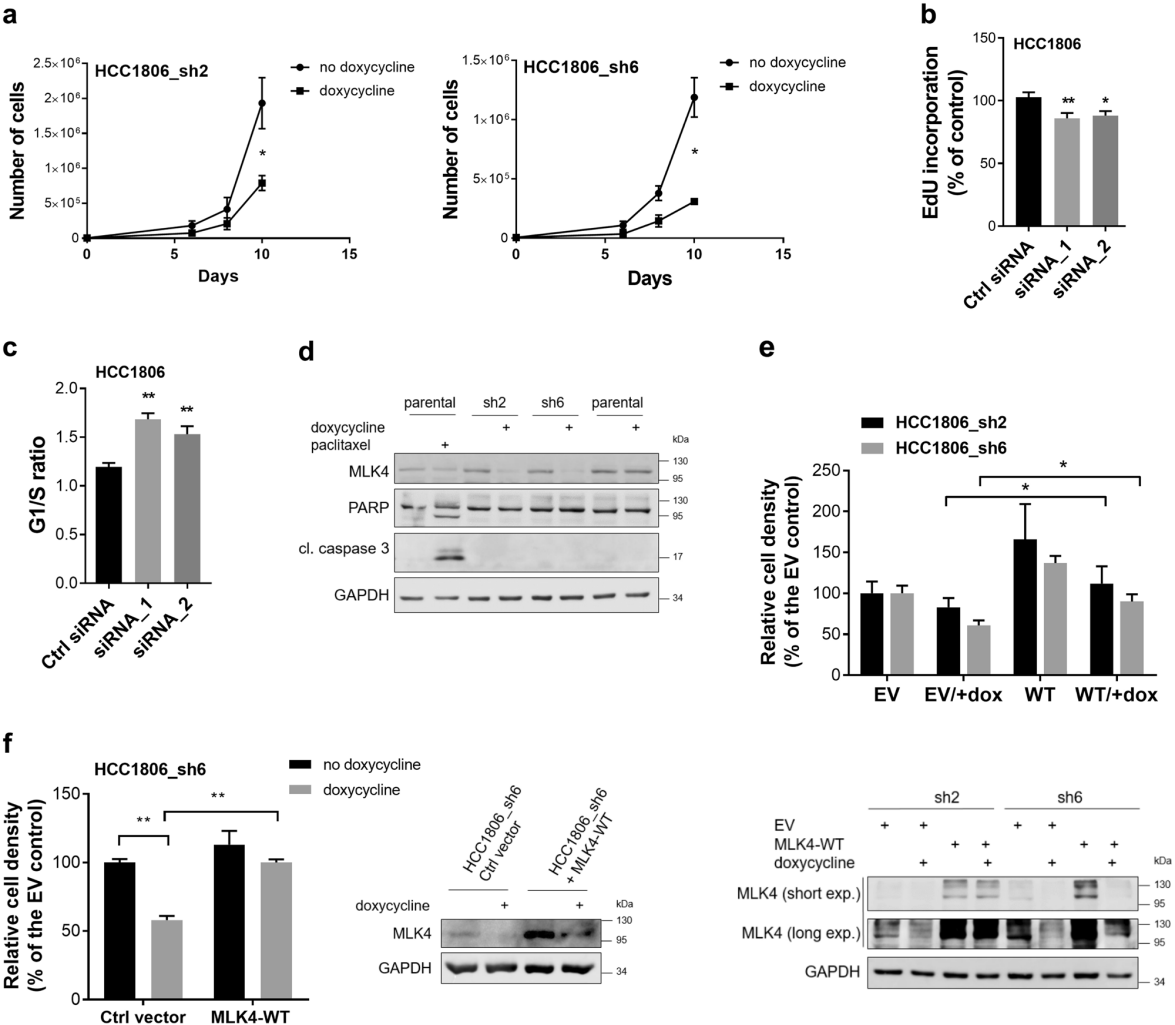
MLK4 depletion leads to cell cycle arrest of TNBC cells with high expression of MLK4. **a** HCC1806_sh2 and HCC1806_sh6 cells were grown with or without doxycycline (1 μg/ml) for 10 days. At day 6, 8 and 10 cells were counted using Bio-Rad Cell Counter TC-20. Error bars indicate ± SEM from three independent experiments (*n* = 3). Statistical comparison of values was performed using the unpaired two-tailed *t*-test. *P < 0.05. **b** Parental HCC1806 cells were transfected with siRNA against MLK4 or control siRNA. After 48 h EdU was added to cells for the next 24 h, then subjected to measurement of EdU incorporation. Error bars indicate ± SEM from at least three independent experiments (*n* = 18). Statistical comparison of values was performed using one-way ANOVA. **P < 0.01, *P < 0.05. **c** 72 h following the transfection of parental HCC1806 with siRNA against MLK4 or control siRNA, cells were harvested and fixed in 70% ethanol. Cells then were treated with RNAse A and stained with propidium iodiode. The distribution of cells in phases of cell cycle by flow cytometry was assessed and the ratio of G1/S was calculated. Error bars indicate ± SEM from four independent experiments (*n* = 4). Statistical comparison of values was performed using one-way ANOVA. **P < 0.01. **d** HCC1806_sh2, HCC1806_sh6 and parental HCC1806 cells were treated with 1 μg/ml doxycycline for 72 h. Whole-cell lysates were analyzed by western blotting. The treatment of parental HCC1806 cells with 5,8 nM paclitaxel for 24 h served as a positive control for apoptosis evidenced with PARP and pro-caspase-3 cleavage. **e**, **f** Reduction in proliferation after MLK4 knock-down is rescued by overexpression of MLK4-WT. **e** HCC1806_sh2 and HCC1806_sh6 were treated with 1 μg/ml doxycycline. Next day cells were transiently transfected with MLK4-WT vector or EV (empty vector) control and were left to grow with or without doxycycline. After 72 h cells were stained with crystal violet and results were quantified by absorbance. Error bars indicate ± SEM from three independent experiments (*n* = 3). Statistical comparison of values was performed using the two-way ANOVA. *P < 0.05. Representative immunoblots showing the level of MLK4-WT in samples lysed after 72 h of transfection are shown below. **f** HCC1806_sh6 with stable overexpression of MLK4-WT and HCC1806_sh6 control vector cells were seeded at low density and were grown with or without 1 μg/ml doxycycline. After 10 days, cells were stained with crystal violet, and quantified by absorbance. Error bars indicate ± SEM from four independent experiments (*n* = 4). Statistical comparison of values was performed using the two-way ANOVA. **P < 0.01. Representative immunoblots showing the level of MLK4 in samples lysed after 96 h doxycycline treatment are shown on the right

To determine whether the observed MLK4 silencing effects are due to inhibition of proliferation or cell death, we counted HCC1806_sh2 and HCC1806_sh6 cells grown with or without doxycycline. We found a significant reduction in the number of cells when MLK4 was depleted (Fig. 3a), but no reduction in cell number for parental HCC1806 cells treated with doxycycline (Supplementary Fig. 4). However, the percentage of viable cells stained with trypan blue was not affected by MLK4 knock-down ranging from 96 to 100% for all the groups regardless of doxycycline treatment which suggests lack of cell death. We next performed EdU incorporation assay and DNA quantification with propidium iodide to measure cell proliferation and to evaluate effects on cell cycle, respectively. Both experiments were performed on the parental HCC1806 cell line in which MLK4 was transiently knocked-down using siRNA (Supplementary Fig. 5a), because the simultaneous doxycycline-induced expression of shRNA together with turboRFP in inducible knock-down cells was interfering with the fluorescent signal measured in those assays. The results obtained in EdU assay confirmed the reduction of cell proliferation induced by MLK4 knock-down in HCC1806 cells (Fig. 3b). The changes in proliferation rate are likely caused by G1/S cell cycle arrest, as indicated by the increase in the G1/S ratio upon MLK4 downregulation (Fig. 3c and Supplementary Fig. 5b). Furthermore, MLK4 knock-down in HCC1806 did not induce apoptosis as there was no increase in apoptotic markers, including cleaved caspase 3 and cleaved PARP (Fig. 3d). To confirm the effects of MLK4 depletion were specifically due to loss of MLK4 expression, rather than off-target effects from the shRNA, we demonstrated that transient or stable overexpression of MLK4-WT, in the inducible MLK4 knock-down HCC1806 cells was sufficient to rescue the reduction of cell growth in a colony formation assay in cells depleted of MLK4 (Fig. 3e, f). These data provide compelling evidence that MLK4 regulates TNBC cells proliferation.

### MLK4 is required for breast cancer cell migration, invasion, 3D growth in vitro and tumor growth in vivo

**Fig. 4:**
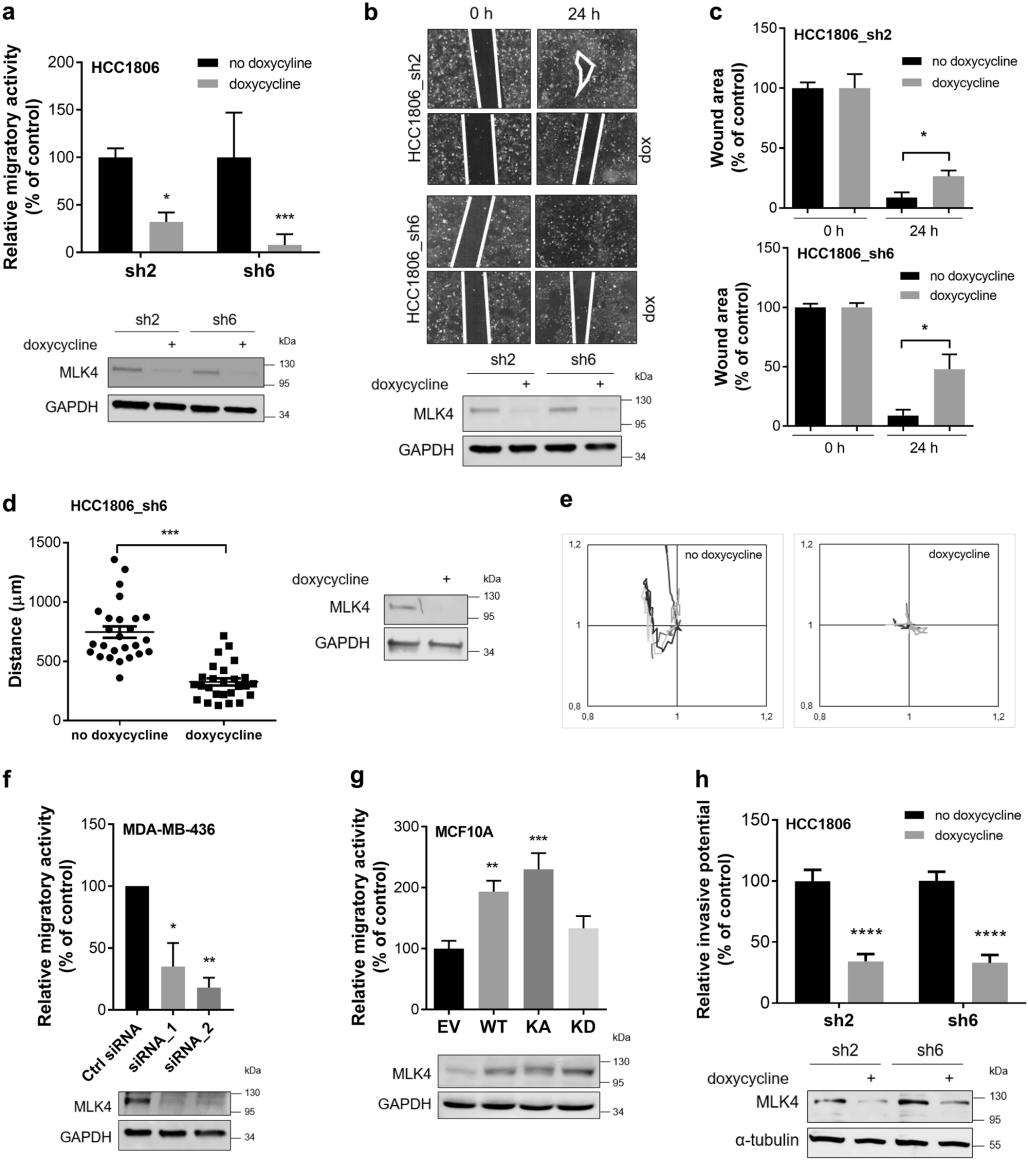
Depletion of MLK4 attenuates migratory and invasive properties of cells. **a** HCC1806_sh2 and HCC1806_sh6 cells were treated with 1 μg/ml doxycycline for at least 72 h, then cells were serum-starved and seeded on transwell inserts. The cells were allowed to migrate for no longer than 24 h. Cells at the bottom of inserts were stained with crystal violet. Five pictures of every condition were taken. Analysis was performed using ImageJ. Error bars indicate ± SEM from three independent experiments (*n* = 15). Statistical analysis was done using unpaired two-tailed *t*-test. ***P < 0.001, *P < 0.05. Below, representative immunoblots showing the level of MLK4 knock-down. **b**, **c** HCC1806_sh2 and HCC1806_sh6 cells were treated with 1 μg/ml doxycycline for at least 72 h and then were subjected to wound-healing assay. Four pictures were taken at each condition. Representative pictures and immunoblots showing the level of MLK4 knock-down are shown (**b**). Quantification was performed using ImageJ (**c**). Error bars indicate ± SEM from four independent experiments (*n* = 12). Statistical comparison of values was performed using the unpaired two-tailed *t*-test. *P < 0.05. **d**, **e** HCC1806_sh6 cells were treated with 1 μg/ml doxycycline for 72 h. Next, single cell migration was monitored for 20 h. Analysis was performed using ImageJ (**d**). Error bars indicate ± SEM (*n* = 28). Statistical comparison of values was performed using the unpaired two-tailed *t*-test. ***P < 0.001. Representative immunoblots showing the level of MLK4 knock-down are shown on the right. Representative tracks of five randomly chosen cells (**e**). The intersection of the *x*- and *y*-axes was taken as the starting point of each cell path to which other points of tracks were normalized. **f** MDA-MB-436 cells were transfected with siRNA against MLK4 or control siRNA. After 48 h, cells were serum-starved and seeded on transwell inserts. The cells were allowed to migrate for no longer than 24 h. Cells at the bottom of inserts were stained with crystal violet. Five pictures of every condition were taken. Analysis was performed using ImageJ. Error bars indicate ± SEM from three independent experiments (n = 15). Statistical analysis was done using one-way ANOVA. **P < 0.01, *P < 0.05. Below, representative immunoblots showing the level of MLK4 knock-down. **g** MCF10A cells were transfected with EV (empty vector) control, MLK4-WT, MLK4-KA (kinase active) and MLK4-KD (kinase dead). After 48 h, cells were serum-starved and seeded on transwell inserts. The cells were allowed to migrate for no longer than 24 h. Cells at the bottom of inserts were stained with crystal violet. Five pictures of every condition were taken. Analysis was performed using ImageJ. Error bars indicate ± SEM from three independent experiments (*n* = 15). Statistical analysis was done using one-way ANOVA. **P < 0.01, ***P < 0.001. Below, representative immunoblots showing the level of MLK4 overexpression. **h** HCC1806_sh2 and HCC1806_sh6 cells were treated with 1 μg/ml doxycycline for at least 72 h, then cells were serum-starved and seeded on transwell inserts coated with Matrigel. The cells were allowed to invade for no longer than 24 h. Cells were stained with crystal violet. Five pictures of every condition were taken. Analysis was performed using ImageJ. Error bars indicate ± SEM from three independent experiments (*n* = 15). Statistical analysis was done using unpaired two-tailed *t*-test. ****P < 0.0001. Below, representative immunoblots showing the level of MLK4 knock-down

Next, we sought to determine the role of MLK4 in migration and invasion of triple-negative breast cancer cells as these processes are required for early steps of metastasis [24]. To test the migratory activity of HCC1806 with doxycycline-inducible MLK4 knock-down, we performed a transwell migration assay in which we observed that MLK4 depletion significantly reduced migration of these cells (Fig. 4a). As an alternative method to investigate migration, we performed a wound-healing assay. Knock-down of MLK4 impaired the ability of cells to close the wound, while the cells not treated with doxycycline closed the wound almost completely (Fig. 4b, c). To strengthen our data we monitored random single cell motility upon the knock-down of MLK4 in HCC1806_sh6 and we found significant decrease in the distance of migration of single cells over the duration of the assay (Fig. 4d, e). Furthermore, we assessed the migration of high-MLK4 MDA-MB-436 TNBC cell line (Fig. 2a, b), and we found that migratory potential of the cells was significantly reduced upon MLK4 depletion (Fig. 4f). We then assessed if overexpression of MLK4 would change the migration rate of control epithelial MCF10A cells and BT474, a poorly invasive breast cancer cell line that is characterized by low MLK4 expression (Fig. 2a, b). We observed that overexpression of MLK4 in both cell lines resulted in a significant increase in cell migration (Fig. 4g and Supplementary Fig. 6). The observed effect is likely kinase-dependent as overexpression of MLK4 kinase dead (KD) mutant [10] did not increase migration rate of MCF10A cells compared to cells expressing MLK4-WT and kinase active (KA) (Fig. 4g). Furthermore, we tested the potential of HCC1806 cells to invade through Matrigel in invasion assay and we found that it was significantly decreased in cells depleted of MLK4, indicating that MLK4 plays an important role in regulation of these processes (Fig. 4h). In addition, we found no differences between migratory or invasive potential of parental HCC1806 cells grown in the presence or absence of doxycycline suggesting that the observed effects are specific to MLK4 knock-down, not to the doxycycline exposure (Supplementary Fig. 7a–c).

**Fig. 5:**
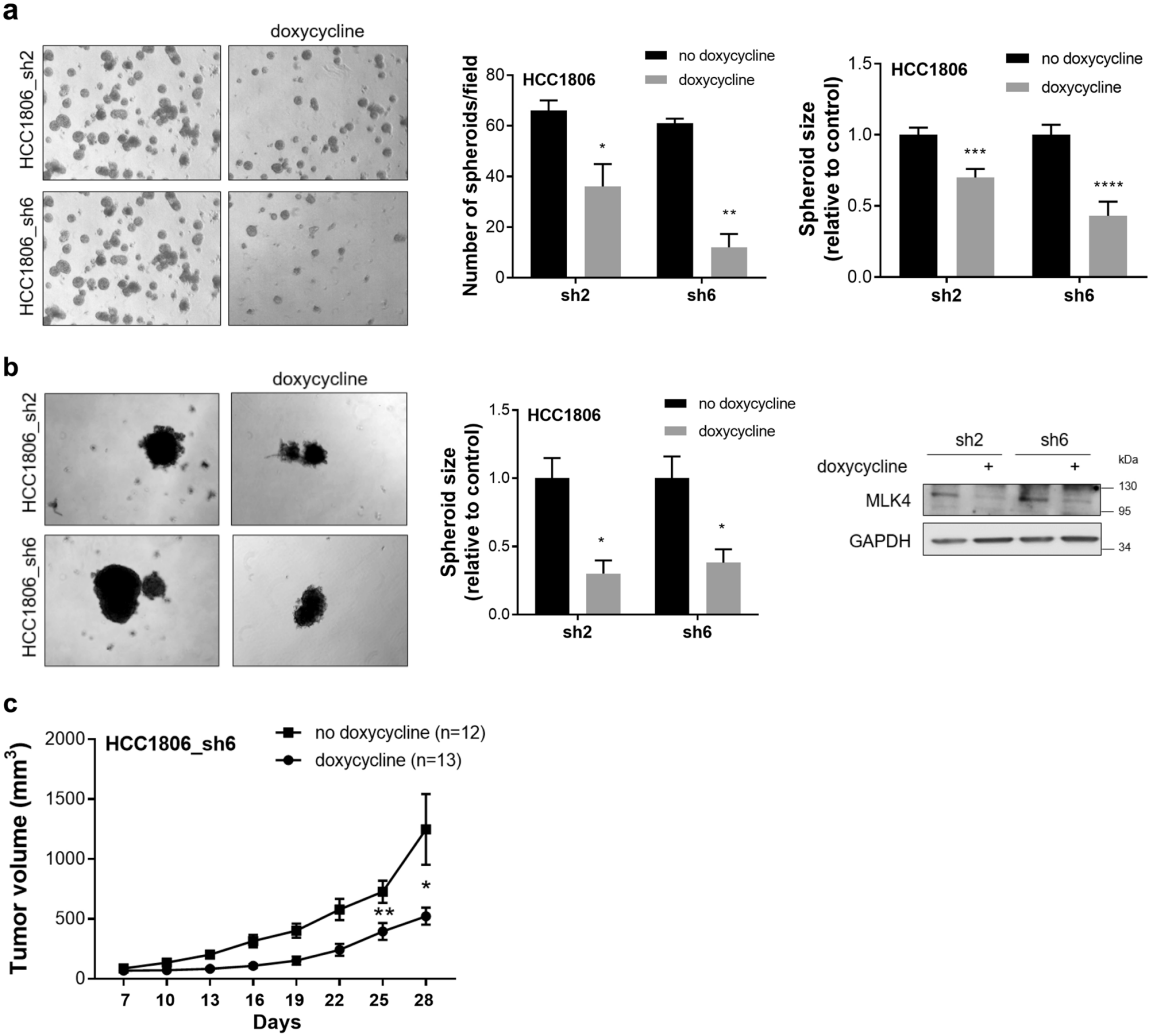
MLK4 knock-down impairs spheroid formation in 3D model in vitro and tumor growth in vivo. **a** HCC1806_sh2 and HCC1806_sh6 cells were seeded on plates coated with Matrigel. Cells were grown for 10 days with or without 1 μg/ml doxycycline. Pictures were taken and the number and size of spheroids were quantified in ImageJ. Error bars indicate ± SEM from three independent experiments. Statistical comparison of values was performed using the unpaired two-tailed *t*-test. ****P < 0.0001, ***P < 0.001, **P < 0.01, *P < 0.05. **b** HCC1806_sh2 and HCC1806_sh6 cells were seeded on low cell attachment surface plates. Cells were grown with or without 1 μg/ml doxycycline. Pictures were taken on day 10 and size of spheroids was quantified in ImageJ. Error bars indicate ± SEM from three independent experiments. Statistical comparison of values was performed using the unpaired two-tailed *t*-test. *P < 0.05. **c** HCC1806_sh6 cells were injected into mammary fat pads of RAG2^−/−^ mice. Doxycycline induction of MLK4 knock-down started one day after the injection. Tumors were measured twice a week. Error bars indicate ± SEM (*n* = 12 for control group and *n* = 13 for doxycycline-treated group). Statistical comparison of values was performed using the unpaired two-tailed *t*-test. **P < 0.01, *P < 0.05

In order to test the role of MLK4 under conditions more closely reflecting the tumor growth in vivo [25, 26], we grew HCC1806 in 3D culture on Matrigel-coated plates. We compared the growth of spheroids formed by HCC1806_sh2 and HCC1806_sh6 cells, treated with or without doxycycline, and we found that MLK4 knock-down caused a significant reduction in the number and size of spheroids (Fig. 5a). Similarly, the formation of spheroids after MLK4 depletion was compromised for HCC1806 cells grown on low cell attachment surface plates (Fig. 5b). The spheroid formation of parental HCC1806 cells was not changed upon treatment with doxycycline (Supplementary Fig. 7d). To assess the importance of MLK4 on tumor growth in vivo, we injected HCC1806_sh6 into mammary fat pads of immune-deficient mice, and induced the knock-down of MLK4 by doxycycline administration. We observed that depletion of MLK4 significantly suppressed tumor growth in this xenograft mouse model (Fig. 5c). Moreover, our control in vivo experiment using parental HCC1806 cells showed no effect of doxycycline administration itself on the tumor growth (Supplementary Fig. 7e). Taken together, our results indicate that high expression of MLK4 promotes cell growth in 3D conditions, leads to increased migratory and invasive behavior of cells, and enhances tumor growth in vivo.

### Upregulation of MLK4 regulates NF-κB signaling and mesenchymal phenotype of breast cancer cells

**Fig. 6:**
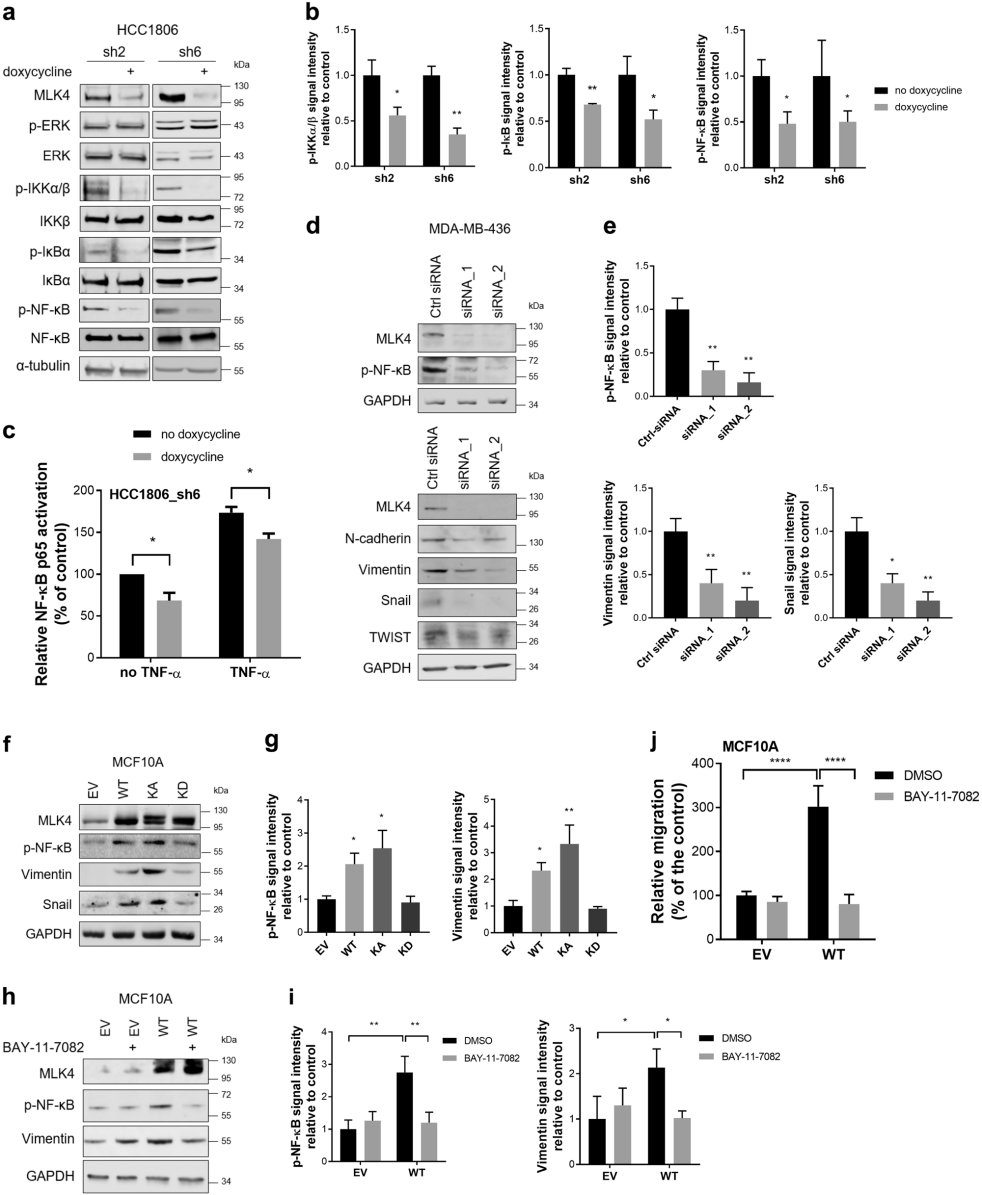
MLK4 activates NF-κB pathway and promotes mesenchymal phenotype of breast cancer cells. **a**, **b** HCC1806_sh2 and HCC1806_sh6 cells were treated with 1 μg/ml doxycycline. Whole cell lysates were analyzed by western blotting (**a**). Quantification analysis of immunoblots was performed using ImageJ (**b**). Error bars indicate ± SEM from four independent experiments (*n* = 4). Statistical comparison of values was performed using the unpaired two-tailed *t*-test. **P < 0.01, *P < 0.05. **c** HCC1806_sh6 cells were treated with 1 μg/ml doxycycline for 72 h, and then were stimulated with 20 ng/ml TNF-α for 1 h, lysed and subjected to TransAM DNA binding assay. Error bars indicate ± SEM from at least three independent experiments (*n* = 3). Statistical comparison of values was performed using the unpaired two-tailed *t*-test. *P < 0.05. **d**, **e** MDA-MB-436 cells were transfected with siRNA against MLK4 or control siRNA. After 72 h whole-cell lysates were analyzed by western blotting (**d**). Quantification analysis of immunoblots was performed using ImageJ (**e**). Error bars indicate ± SEM from three independent experiments (*n* = 3). Statistical comparison of values was performed using one-way ANOVA. **P < 0.01, *P < 0.05. **f**, **g** MCF10A cells were transfected with EV (empty vector) control, MLK4-WT, MLK4-KA (kinase active) and MLK4-KD (kinase dead). After 48 h whole-cell lysates were analyzed by western blotting (**f**). Quantification analysis of immunoblots was performed using ImageJ (**g**). Error bars indicate ± SEM from three independent experiments (*n* = 3). Statistical comparison of values was performed using one-way ANOVA. **P < 0.01, *P < 0.05. **h**, **i** MCF10A cells were transfected with EV (empty vector) control or MLK4-WT. After 8 h medium was changed and cells were treated with 10 µM BAY-11-7082 or DMSO for 48 h. Whole cell lysates were analyzed by western blotting (**h**). Quantification analysis of immunoblots was performed using ImageJ (**i**). Error bars indicate ± SEM from three independent experiments (n = 3). Statistical comparison of values was performed using two-way ANOVA. **P < 0.01, *P < 0.05. **j** MCF10A cells were transfected with EV (empty vector) control or MLK4-WT. After 48 h cells were seeded on transwell inserts. When cells attached to the bottom of inserts, the medium was changed and cells were treated with 10 µM BAY-11-7082 or DMSO overnight. Cells that migrated through the inserts were stained with crystal violet. Five pictures of every condition were taken. Analysis was performed from three independent experiments using ImageJ (*n* = 15). Statistical analysis was done using two-way ANOVA. ****P < 0.0001

In an effort to define the mechanisms behind the observed effects we investigated the signaling pathways regulated by MLK4. To assess the MAPK pathways potentially altered by MLK4 depletion we utilized a phospho-MAPK array using HCC1806_sh6 cell line. We observed that there were no significant changes in phosphorylation of the ERK, JNK, or p38 pathways following MLK4 silencing (Supplementary Fig. 8a and b). Therefore, we tested other possible pathways and we observed that MLK4 depletion led to a drop in phosphorylation of IKKα/β, IκB and NF-κB (Fig. 6a, b). In order to further investigate the role of MLK4 in NF-κB activation we stimulated HCC1806_sh2 and HCC1806_sh6 cells with TNF-α, which is one of the most potent inducers of NF-κB pathway (Supplementary Fig. 9a and b). MLK4 knock-down resulted in decreased phosphorylation of NF-κB at 5 and 10 min after TNF-α stimulation, but there were no differences at 25 min, indicating that the most significant involvement of MLK4 in activation of NF-κB pathway takes place at early stages of pathway activity (Supplementary Fig. 9a and b). To validate NF-κB as an important downstream target of MLK4, we used the ELISA-based TransAM assay to quantify NF-κB-p65 DNA binding activity. Doxycycline-induced depletion of MLK4 significantly reduced active NF-κB p65 DNA binding in both unstimulated and TNF-α-stimulated cells, which provides additional evidence that MLK4 is a regulator of NF-κB signaling in TNBC cells (Fig. 6c). The same experiments performed on parental HCC1806 cells revealed no doxycycline-dependent changes in the NF-κB activation (Supplementary Fig. 10a and b). Next, we analyzed the expression of NF-κB target genes in HCC1806_sh6 cells. After treating cells with TNF-α to induce NF-κB activation, we observed that the expression of NF-κB target genes, including several cytokines and transcription factors, was markedly downregulated upon MLK4 knock-down (Supplementary Fig. 11). These results demonstrate that the loss of MLK4 leads to reduced NF-κB signaling along with impaired induction of NF-κB target genes by pro-inflammatory cytokine TNF-α in TNBC cells.

Recent studies show that the activation of NF-κB pathway has been associated with a regulation of EMT and increased metastatic potential in breast cancer [27–31]. To test the effects of MLK4 on the maintenance of the mesenchymal phenotype, we used high-MLK4 mesenchymal MDA-MB-436 cells. The depletion of MLK4 in this cell line led to the downregulation of mesenchymal markers and reduction of NF-κB phosphorylation (Fig. 6d, e). Conversely, overexpression of MLK4 in low-MLK4 cell lines (MCF10A and BT474) resulted in the upregulation of mesenchymal markers and increased phosphorylation of NF-κB (Fig. 6f, g and Supplementary Fig. 12). In accordance with these findings, gene set enrichment analysis (GSEA) run on TCGA and GSE76275 datasets indicated a positive correlation between MLK4 mRNA levels and EMT-related genes expression in clinical specimens (Supplementary Fig. 13).

We then evaluated if phenotypic changes induced by MLK4 overexpression or knock-down in breast cancer cell lines were directly controlled by NF-κB signaling. We first observed that blocking NF-κB pathway activity with a small-molecule inhibitor, BAY-11-7082 [32], in parental HCC1806 cells led to a significant reduction in anchorage-dependent colony formation and the migratory potential of these cells, which phenocopied the effects obtained in HCC1806-inducible MLK4 knock-down cells (Supplementary Fig. 14a and b). Additionally, we found that inhibition of NF-κB pathway with BAY-11-7082 in MCF10A cells overexpressing MLK4-WT significantly decreased the expression of the mesenchymal marker Vimentin (Fig. 6h, i), and reduced migration of those cells (Fig. 6j). Collectively, our data indicate that MLK4 contributes to the acquisition of the mesenchymal phenotype and increased migratory potential of breast cancer cells through NF-κB-dependent mechanism.

### High MLK4 expression is associated with an increased metastatic potential of tumors in TNBC patients

**Fig. 7:**
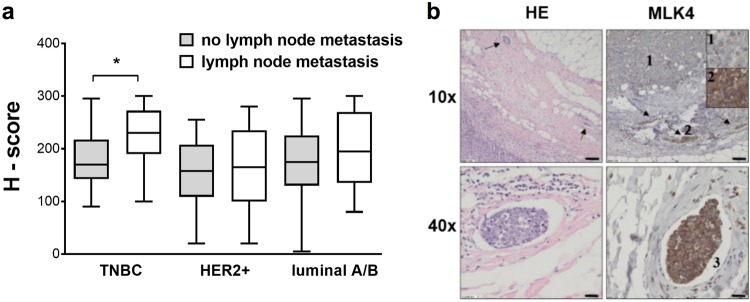
High MLK4 expression is associated with lymph nodes metastasis in TNBC patients. **a** Immunohistochemical staining for MLK4 was performed in paraffin-embedded blocks of tumor tissues of 129 breast cancer samples (TNBC – 46, HER2-positive – 26, luminal A – 26, luminal B – 31). The staining was evaluated using the H-score system. Information on occurrence of lymph node metastasis was collected from pathologic reports. The intensity of immunohistochemical staining expressed by H-score was calculated using Mann–Whitney *U* test. *P < 0.05. **b** NST (no specific type) of triple-negative carcinoma showing lymphovascular invasion (arrows) in hematoxylin eosin staining and strong MLK4 staining in the intravascular component (2, 3) comparing to the main bulk of the tumor (1). Scale bar 100 µm (×10 magnification) and 20 µm (×40 magnification)

To translate our results into a more clinically relevant setting, we performed an immunohistochemical staining for MLK4 of 129 breast cancer patients, including 46 diagnosed with TNBC (Supplementary Table 1). All of the samples showed some degree of positive MLK4 cytoplasmic staining as shown in representative pictures (Supplementary Fig. 15); however; the intensity of the staining was stronger in TNBC samples compared to other subtypes of breast cancer (Fig. 7a), which corresponds to our transcriptomic data (Fig. 1b, e). Moreover, more intense staining of TNBC samples significantly correlated with the occurrence of lymph node metastasis (Fig. 7a). Interestingly, pictures of cases with lymphovascular invasion showed that MLK4 staining was stronger in intravascular component compared to the main bulk of the tumor (Fig. 7b). These data indicate that high MLK4 expression drives aggressiveness and enhances the metastatic potential of TNBC.

## Discussion

Despite an increasing number of studies describing the involvement of MLKs in tumorigenesis, the role of MLK4 in cancer progression is still relatively unknown and, what’s more, seems complex and often contradictory. Early reports suggested that MLK4 does not have the ability to activate ERK, JNK and p38 pathways and that it negatively regulates MAPK and TLR4 signaling [11, 12]. In agreement with these reports, Blessing et al. have recently showed that MLK4 interacts with MLK3 limiting its kinase activity and downstream MAPK pathways in ovarian cancer [13]. However, the lack of kinetic activity of MLK4 was not confirmed by other studies where MLK4 was shown to activate the JNK and ERK pathways [10, 14, 15]. Direct phosphorylation of MEK1/2 by MLK4, and other MLKs, was shown to promote resistance to RAF inhibitors in melanoma, establishing a pro-tumorigenic role of MLK4 [10]. A recent study also demonstrated that MLK4 is involved in the regulation of NF-κB pathway by direct phosphorylation of IKKα/β in glioma cells, which further defines a tumor-promoting role of MLK4 in certain cancer types [16]. The role of MLK4 in colon cancer has also been studied due to multiple point mutations identified in MLK4 in primary tumors and colon cancer cell lines [14, 15, 33]. The functional consequences of those mutations were described in our previous research where we identified them as loss-of-function mutations and defined a tumor suppressive role of MLK4 in colon cancer [15]. On the contrary, a different study by Martini et al. described the same MLK4 mutations as oncogenic [14]. In the light of those controversial reports, it is clear that further investigation of MLK4 in tumorigenesis is needed. Nevertheless, these studies show the complexity of MLK4 signaling pathways in different types of cancer and highlight a multitude of downstream substrates that MLK4 will regulate.

Here, we studied the functional outcomes of MLK4 upregulation on the progression of breast cancer and its contribution to the aggressive phenotype of breast cancer cells. We demonstrated that MLK4 expression was upregulated in breast cancer, mostly in TNBC, and was required for proliferation and clonogenic potential of cells expressing high endogenous levels of MLK4. The depletion of MLK4 caused reduction in anchorage-dependent growth not only in TNBC cell lines, but also in non-TNBC cell line, ZR-75-1 and MCF7 that were characterized by increased MLK4 expression and abundance. This might indicate a more general mechanism of action that is not dependent on breast cancer subtypes, but rather directly on the expression levels of MLK4. However, major amplification of MLK4 was identified in patients with triple-negative breast invasive carcinoma and the lack of targeted therapies in TNBC prompt us to investigate the role of MLK4 in this specific subtype of breast cancer. We found the most striking effects after silencing of MLK4 on migration and invasion of TNBC cells. Furthermore, the ability of cells to grow in 3D cell culture and form tumors in vivo was decreased by MLK4 knock-down. We also investigated downstream pathways activated by MLK4 in TNBC cells and found that the transcriptional activity of NF-κB was reduced upon the depletion of MLK4 in TNBC cells. Previous studies have demonstrated that NF-κB can influence mesenchymal phenotype of breast cancer cells by activation of EMT regulatory factors, including Snail, Slug, TWIST and Sip1 [27, 29]. It has also been shown that NF-κB stabilizes Snail which triggers increased migration and invasion of cancer cells [34]. Here, we demonstrated that knock-down of MLK4 reduced the expression of mesenchymal markers. Conversely, overexpression of MLK4 promoted the expression of mesenchymal markers. As EMT is accompanied by changes in the metastatic potential of cells, we observed that overexpression of MLK4 increased the migration rate of cells. In contrast, MLK4 depletion had the opposite effect, as we observed a reduction of migratory and spheroid-forming potential of the cells. Moreover, immunohistochemical staining of samples obtained from breast cancer patients revealed a significant correlation between high MLK4 expression and occurrence of metastasis, which strengthens our in vitro data on TNBC cell lines and implicates the importance of MLK4 in processes responsible for tumor aggressiveness such as migration and invasion.

Epithelial–mesenchymal transition drives carcinoma progression and plays an immense role in metastasis [35, 36]. The acquisition of migratory phenotypes is a crucial step in the metastatic processes; thus deciphering the mechanisms responsible for the invasive properties of tumor cells is crucial for the development of novel clinical strategies [35, 37]. Our study draws attention to upregulation of MLK4 and the role of its aberrant activity in breast cancer, predominantly in driving migration and invasion. We provide the evidence that high expression of MLK4 leads to activation of NF-κB pathway, promotes invasiveness and expression of mesenchymal markers, and contributes to a malignant phenotype of TNBC cells. In conclusion, our data indicate that MLK4 might constitute a potential novel target in personalized therapy for a subset of breast cancer patients.

## Materials and methods

### Cell lines

HCC1806, MDA-MB-436, HCC1395, ZR-75-1, T47D, MCF7, BT474, and MCF10A were a kind gift from CRUK Manchester Institute and they were authenticated by short tandem repeat profiling by CRUK MI core facility unit. HCC1806 was authenticated again in August 2017 by ATCC Service. HCC1569 and HCC1599 were purchased from ATCC in April 2016. MDA-MB-453 and CAL-85-1 were purchased from DSMZ in November 2014 and November 2016, respectively. MDA-MB-321 and BT20 was a kind gift from Department of Immunology, Medical University of Warsaw (purchased from ATCC in February 2018). ATCC and DSMZ authenticate cell lines by short tandem repeat profiling. HCC1806, HCC1395, BT474, BT20, ZR-75-1, T47D, HCC1599 and HCC1569 were cultured in RPMI-1640 supplemented with 10% FCS, 1% penicillin/streptomycin, 2 mM l-glutamine and 1 mM sodium pyruvate. MCF7, CAL-85-1, MDA-MB-453 and MDA-MB-231 were cultured in DMEM supplemented with 10% FCS, 1% penicillin/streptomycin, 2 mM l-glutamine and 1 mM sodium pyruvate. MDA-MB-436 were cultured in RPMI-1640 with 25 mM HEPES supplemented with 10% FCS, 1% penicillin/streptomycin, 2 mM l-glutamine, 1 mM sodium pyruvate and 10 µg/ml insulin. MCF10A were grown in DMEM/F12 supplemented with 5% horse serum, 20 ng/ml EGF, 0.5 mg/ml hydrocortisone, 100 ng/ml cholera toxin, 10 µg/ml insulin, 1% penicillin/streptomycin.

### Generation of doxycycline-inducible cell lines

Parental HCC1806 and HCC1599 were used to generate cells with doxycycline-inducible knock-down of MLK4. HEK293T cells were transfected with human TRIPZ shRNA against MLK4 (Dharmacon) to generate a lentiviral stock. The sequences of shRNA are as follows: sh2_ATCAGAATGTTAAGTTCCC and sh6_TCTTGATACACTACAATCA. Cells were transduced with lentiviral stocks and cell lines generated by antibiotic selection with puromycin. Doxycycline (Sigma) in 1 µg/ml concentration was used to induce knock-down of MLK4. Generation of additional cell lines is described in Supplementary Information file.

### Migration and invasion assays

Migration and invasion assays were performed using transwell inserts (Corning) or Matrigel-coated inserts (Corning), respectively. Cells were subjected to MLK4 knock-down or overexpression, then serum-starved, trypsinized and seeded into upper chamber. Chemoattractant in the lower chamber was medium with 20% serum. After no more than 24 h, cells were fixed, stained with crystal violet and five pictures per condition were taken. Quantitative analysis of was performed using ImageJ.

### Wound-healing assay

HCC1806 were grown with or without doxycycline for at least 3 days until they reached the confluence. The monolayer was scratched using a 200 µl pipette tip. Three pictures of every wound in every condition were taken at time points 0 and 24 h. Quantitative analysis was performed using ImageJ.

### 3D cell culture

3D cell culture on Matrigel was performed as described previously [38]. Briefly, 5000 cells were seeded on the top of solidified Matrigel. Growth media with or without 1 µg/ml doxycycline and 2.5% Matrigel was added to the wells and it was replaced every few days. After 10 days pictures were taken and the number of spheroids counted. As an alternative, cells were grown in a low-attachment surface Nunclon Sphera plates (Thermo Fisher Scientific) in medium with or without doxycycline for 10 days.

### Mouse xenografts and in vivo studies

All procedures were approved by Local Ethics Committee at University of Warsaw (372/2017) and carried out in accordance with the requirements of EU (Directive 2010/63/EU) and Polish (Dz. U. poz. 266/15.01.2015) legislation. 8-14-week old RAG2^-/-^ female mice were injected into mammary fat pads with 3 × 10^6^ HCC1806_sh6 cells or parental HCC1806 with Matrigel in proportion 1:1. Mice were allocated randomly into cages and doxycycline was administered in drinking water 1 day after injections. During experiments the mice were kept under specific pathogen free (SPF) conditions in the individually ventilled cages (IVC) at temperature 22 °C (±2 °C), 55 % (±5%) humidity and 13 h/11 h light cycle. All cages were environmentally enriched to improve the well-being of the animals. All animals had ad libitum access to food and water. The experimental groups were blinded—the researchers measuring tumor size were not aware which treatment group they are dealing with. Tumor formation was monitored and tumor volume based on caliper measurements was calculated by the formula: tumor volume = (*D* × *d*^2^ × *π*)/6, where *D* is the bigger measurement, and *d* is smaller measurement. Mice were culled when tumors reached max permitted measurement or after 4 weeks post injection. No mice were excluded from the data analysis for in vivo experiments.

### Statistical analysis

Statistical comparison of values was performed using the unpaired two-tailed *t*-test or ANOVA with Tukey’s multiple-comparison tests as indicated in figure legends. Mann–Whitney *U* test was used for transcriptomic analysis and intensity of immunohistochemical staining expressed by H-score. Calculations were done using GraphPad Prism 7 software and results were considered statistically significant at *P* < 0.05. Sample sizes and statistical significance are indicated in the figure legends. Data are expressed as mean ± SEM. Experiments were performed at least three times independently. The sample size for in vitro experiments was not chosen with consideration of the power needed to detect a pre-specified effect size. The number of mice for in vivo experiments was chosen using algorithm available on http://biomath.info/ using two-tailed unpaired *t*-test and values *α* = 0.05 and 1−*β* = 0.8.

For additional methods, please see Supplementary Information file available at Oncogene’s website.

## Supplementary information

Supplementary Information file

## References

[1] Goldhirsch A., Wood W. C., Coates A. S., Gelber R. D., Thürlimann B., Senn H.-J. (2011). Strategies for subtypes—dealing with the diversity of breast cancer: highlights of the St Gallen International Expert Consensus on the Primary Therapy of Early Breast Cancer 2011. Annals of Oncology.

[2] Yersal O, Barutca S (2014). Biological subtypes of breast cancer: Prognostic and therapeutic implications. World J Clin Oncol [Internet].

[3] Prat Aleix, Pineda Estela, Adamo Barbara, Galván Patricia, Fernández Aranzazu, Gaba Lydia, Díez Marc, Viladot Margarita, Arance Ana, Muñoz Montserrat (2015). Clinical implications of the intrinsic molecular subtypes of breast cancer. The Breast.

[4] Kumar P, Aggarwal R (2016). An overview of triple-negative breast cancer. Arch Gynecol Obstet [Internet].

[5] Kalimutho M, Parsons K, Mittal D, López JA, Srihari S, Khanna KK (2015). Targeted therapies for triple-negative breast cancer: combating a stubborn disease. Trends Pharmacol Sci [Internet].

[6] Haffty BG, Yang Q, Reiss M, Kearney T, Higgins SA, Weidhaas J (2006). Locoregional relapse and distant metastasis in conservatively managed triple negative early-stage breast cancer. J Clin Oncol.

[7] Dent R, Trudeau M, Pritchard KI, Hanna WM, Kahn HK, Sawka CA (2007). Triple-negative breast cancer: clinical features and patterns of recurrence. Clin Cancer Res.

[8] Burstein MD, Tsimelzon A, Poage GM, Covington KR, Contreras A, Fuqua SAW (2015). Comprehensive genomic analysis identifies novel subtypes and targets of triple-negative breast cancer. Clin Cancer Res [Internet].

[9] Gallo KA, Johnson GL (2002). Mixed-lineage kinase control of JNK and p38 MAPK pathways. Nat Rev Mol Cell Biol [Internet].

[10] Marusiak AA, Edwards ZC, Hugo W, Trotter EW, Girotti MR, Stephenson NL (2014). Mixed lineage kinases activate MEK independently of RAF to mediate resistance to RAF inhibitors. Nat Commun [Internet].

[11] Abi Saab WF, Brown MS, Chadee DN (2012). MLK4β functions as a negative regulator of MAPK signaling and cell invasion. Oncog [Internet].

[12] Seit-Nebi A, Cheng W, Xu H, Han J (2012). MLK4 has negative effect on TLR4 signaling. Cell Mol Immunol [Internet].

[13] Blessing NA, Kasturirangan S, Zink EM, Schroyer AL, Chadee DN (2017). Osmotic and heat stress-dependent regulation of MLK4β and MLK3 by the CHIP E3 ligase in ovarian cancer cells. Cell Signal [Internet].

[14] Martini M, Russo M, Lamba S, Vitiello E, Crowley EH, Sassi F (2013). Mixed lineage kinase MLK4 is activated in colorectal cancers where it synergistically cooperates with activated RAS signaling in driving tumorigenesis. Cancer Res [Internet].

[15] Marusiak AA, Stephenson NL, Baik H, Trotter EW, Li Y, Blyth K (2016). Recurrent MLK4 loss-of-function mutations suppress JNK signaling to promote colon tumorigenesis. Cancer Res [Internet].

[16] Kim SH, Ezhilarasan R, Phillips E, Gallego-Perez D, Sparks A, Taylor D (2016). Serine/threonine kinase MLK4 determines mesenchymal identity in glioma stem cells in an NF-κB-dependent manner. Cancer Cell [Internet].

[17] Cerami E, Gao J, Dogrusoz U, Gross BE, Sumer SO, Aksoy BA (2012). The cBio cancer genomics portal: an open platform for exploring multidimensional cancer genomics data. Cancer Discov [Internet].

[18] Ciriello G, Gatza ML, Beck AH, Wilkerson MD, Rhie SK, Pastore A (2015). Comprehensive molecular portraits of invasive lobular breast. Cancer Cell [Internet].

[19] Weinstein JN, Collisson EA, Mills GB, KRM Shaw, Ozenberger BA (2013). The Cancer Genome Atlas Pan-Cancer analysis project. Nat Genet [Internet].

[20] Guedj M, Marisa L, de Reynies A, Orsetti B, Schiappa R, Bibeau F (2012). A refined molecular taxonomy of breast cancer. Oncogene [Internet].

[21] Maubant S, Tesson B, Maire V, Ye M, Rigaill G, Gentien D (2015). Transcriptome analysis of Wnt3a-treated triple-negative breast cancer cells. PLoS One [Internet].

[22] Sabatier R, Finetti P, Cervera N, Lambaudie E, Esterni B, Mamessier E (2011). A gene expression signature identifies two prognostic subgroups of basal breast cancer. Breast Cancer Res Treat [Internet].

[23] Györffy B, Lanczky A, Eklund AC, Denkert C, Budczies J, Li Q (2010). An online survival analysis tool to rapidly assess the effect of 22,277 genes on breast cancer prognosis using microarray data of 1,809 patients. Breast Cancer Res Treat [Internet].

[24] Hanahan D, Weinberg RA (2011). Hallmarks of cancer: the next generation. Cell [Internet].

[25] Breslin S, O’Driscoll L (2013). Three-dimensional cell culture: the missing link in drug discovery. Drug Discov Today [Internet].

[26] Pal A, Kleer CG. Three dimensional cultures: a tool to study normal acinar architecture vs. malignant transformation of breast cells. J Vis Exp [Internet]. 2014. https://www.ncbi.nlm.nih.gov/pubmed/24797513. 10.3791/51311 PMC4145693 24797513

[27] Pires BRB, Mencalha AL, Ferreira GM, de Souza WF, Morgado-Díaz JA, Maia AM (2017). NF-kappaB Is Involved in the Regulation of EMT Genes in Breast Cancer Cells. PLoS One [Internet].

[28] Huber MA, Azoitei N, Baumann B, Grünert S, Sommer A, Pehamberger H (2004). NF-kappaB is essential for epithelial-mesenchymal transition and metastasis in a model of breast cancer progression. J Clin Invest [Internet].

[29] Li CW, Xia W, Huo L, Lim SO, Wu Y, Hsu JL (2012). Epithelial-mesenchymal transition induced by TNF-α requires NF-κB-mediated transcriptional upregulation of Twist1. Cancer Res [Internet].

[30] Huang CY, Hsieh NT, Li CI, Weng YT, Liu HS, Lee MF (2017). MED28 regulates epithelial-mesenchymal transition through NFκB in human breast cancer cells. J Cell Physiol [Internet].

[31] Neil JR, Schiemann WP (2008). Altered TAB1:I kappaB kinase interaction promotes transforming growth factor beta-mediated nuclear factor-kappaB activation during breast cancer progression. Cancer Res [Internet].

[32] Pierce JW, Schoenleber R, Jesmok G, Best J, Moore SA, Collins T (1997). Novel inhibitors of cytokine-induced IkappaBalpha phosphorylation and endothelial cell adhesion molecule expression show anti-inflammatory effects in vivo. J Biol Chem [Internet].

[33] Bardelli A, Parsons DW, Silliman N, Ptak J, Szabo S, Saha S (2003). Mutational analysis of the tyrosine kinome in colorectal cancers. Sci [Internet].

[34] Wu Y, Deng J, Rychahou PG, Qiu S, Evers BM, Zhou BP (2009). Stabilization of snail by NF-kappaB is required for inflammation-induced cell migration and invasion. Cancer Cell [Internet].

[35] Sleeman J, Steeg PS (2010). Cancer metastasis as a therapeutic target. Eur J Cancer [Internet].

[36] Thiery JP, Acloque H, Huang RYJ, Nieto MA (2009). Epithelial-mesenchymal transitions in development and disease. Cell [Internet].

[37] Gandalovičová A, Rosel D, Fernandes M, Veselý P, Heneberg P, Čermák V (2017). Migrastatics-anti-metastatic and anti-invasion drugs: promises and challenges. Trends Cancer [Internet].

[38] Lee GY, Kenny PA, Lee EH, Bissell MJ (2007). Three-dimensional culture models of normal and malignant breast epithelial cells. Nat Methods [Internet].

