# Supplementary material for: Upregulation of MLK4 promotes migratory and invasive potential of breast cancer cells
Source: Oncogene. 2018 Dec 14;38(15):2860–75. doi: 10.1038/s41388-018-0618-0 (PMC6484767; doi:10.1038/s41388-018-0618-0)
Supplement: Supplementary file 1 — Supplementary Information file [file 41388_2018_618_MOESM1_ESM.doc]

**Supplementary Information**

**Upregulation of MLK4 promotes migratory and invasive potential of breast cancer cells**

Anna A. Marusiak, Monika K. Prelowska, Dawid Mehlich, Michal Lazniewski, Klaudia Kaminska, Adam Gorczynski, Aleksandra Korwat, Olga Sokolowska, Hanna Kedzierska, Jakub Golab, Wojciech Biernat, Dariusz Plewczynski, John Brognard, Dominika Nowis

**Content:**

1. **Supplementary Methods**
2. **Supplementary Figure Legends and Figures 1-15**
3. **Supplementary Table 1**
4. **Supplementary References**

**Supplementary Methods**

***Gene expression analysis***

Gene expression data were obtained from Array Express, a public repository of  genomic data, and include samples from Cartes d'Identite des Tumeurs project (with 537 patients analyzed using Affymetrix HG-U133 Plus 2,0 GeneChips array; Array Express identifier E-MTAB-365); Institute Curie (Maire cohort, 356 patients; E-GEOD-65216) and samples from Institute Paoli-Calmettes (266 patients, E-GEOD-21653) (1–3). Samples were assigned to specific subgroups of breast cancer based on immunohistochemical (IHC) status of estrogen receptor (ER), progesterone receptor (PR) and receptor tyrosine-protein kinase erbB-2 (HER2). The triple negative breast cancer group constitutes samples with no expression of either receptor, for HER2+ group only expression of HER2 receptor was present with no expression for ER or PR. The ER/PR+ subgroup is composed of samples where either ER or PR receptor were expressed. The quality of each microarray was analyzed using arrayQualityMetrics package (4) and assessed using following metrics: (I)  the absolute value of M (log ratio), (II) the mean and interquartile range of M, (III) the mean absolute difference of M and (IV) Normalized Unscaled Standard Error. An array was classified as an outlier if it was detected as such using at least two of the abovementioned metrics. Each study was analyzed independently. Gene expression analysis was carried using several R packages available within Bioconductor framework (5). After the removal of the outliers, the raw CEL files were processed using gcrma algorithm *P*-values between selected distributions and were calculated using Mann–Whitney U test. Differential gene expression (DE) was carried out using linear model implemented in limma package (6) with adjustment for unknown batch effects performed according to the vignette of the ‘sva’ package (7). For this analysis, 40% of probes with the lowest variance across all the samples were excluded. The number of surrogate variables was estimated using asymptotic approach proposed by Leek. *P*-values were adjusted for multiple testing by using the method of Benjamini and Hochberg to control the false discovery rate. Results were visualized using ggplot2 package.

***Code availability***

All the scripts used for the transcriptomic analysis are available publicly ([https://bitbucket.org/4dnucleome/brest_cancer_analysis/](https://www.google.com/url?q=https%3A%2F%2Fbitbucket.org%2F4dnucleome%2Fbrest_cancer_analysis%2F&sa=D&sntz=1&usg=AFQjCNE0vBtG9Mphb9VblLDcJaVx4_uJ_A)). Additionally, we provide information about our classification of samples to specific breast cancer subtypes.

***Reagents***

Recombinant human Tumor Necrosis Factor-α (TNF-α) was purchased from Sigma Aldrich, dissolved in PBS, stored in -80^0^C and used at final concentration of 20 ng/ml. BAY-11-7082 was purchased from Absource Diagnostics GmbH, dissolved in DMSO, stored at -80^0^C and used at final concentration of 10 µM.

***Generation of doxycycline-inducible cell lines***

For doxycycline-inducible overexpression of MLK4-WT in BT474 cell line, we used MLK4-WT plasmid (cloned into pLenti6/TO/V5-DEST vector) and pLenti3.3/TR (for tetracycline repressor expression) to generate lentiviral stocks in HEK293T cells. BT474 were transduced with lentiviral stocks and cell line generated by antibiotic selection (Blasticidin (Invitrogen) and Geneticin (Gibco)). Doxycycline (Sigma) in 1 µg/ml concentration was used to induce overexpression of MLK4. For stable overexpression of MLK4-WT in HCC1806_sh6 cell line, we used MLK4-WT plasmid (cloned into pLenti6/TO/V5-DEST vector) or Ctrl vector (empty pLenti6/TO/V5-DEST vector) to generate lentiviral stocks in HEK293T cells. HCC1806_sh6 were transduced with lentiviral stocks and cell lines (HCC1806_sh6 MLK4-WT and HCC1806_sh6 Ctrl vector) were generated by antibiotic selection (Blasticidin (Invitrogen)).

***Plasmids and transfection***

MLK4-WT was obtained from GeneCopoeia, cloned and mutations (KA=kinase active and KD=kinase dead) were introduced as previously described (8). JetPRIME (Polyplus Transfections) or GeneJuice (Millipore) were used for transient overexpression studies. DharmaFECT1 (Dharmacon) or JetPRIME were used for siRNA transfections. siRNA against MLK4 (5’-GGAAAGAUGCUCAGAGAGAUU-3’ and 5’-AGGAGAAGCCCAAGGUAAAUU-3’) and control non-targeting siRNA were purchased from Thermo Fisher Scientific.

***Protein lysate preparation and western blotting***

Cells were lysed with Triton X-100 (Cell Signaling Technology) or RIPA (Sigma) lysis buffers supplemented with protease and phosphatase inhibitor tablets (Roche). Proteins were resolved by SDS-PAGE and analyzed by western blotting. Primary antibodies: phospho-IKKα/β (Ser176/177) (C84E11) #2078, IKKβ (D30C6) #8943, phospho-IκBα (Ser32) (14D4) #2859, IκBα (L35A5) #4814, phospho-NF-κB (S536) (93H1) #3033, NF-κB (D14E12) #8242, Vimentin (D21H3) #5741, E-cadherin (24E10) #3195, N-cadherin (D4R1H) #13116, Snail (C15D3) #3879, TWIST1 #46702, GAPDH #2118, p-ERK1/2 (T202/Y204) (197GE) #4377, ERK (137F5) #4695, PARP (46D11) #9532, cleaved caspase 3 (D175) #96645, tubulin (DM1A) #12351 (Cell Signaling Technology); MLK4β (A302-610A) (Bethyl Laboratories). Quantification of immunoblots was performed using ImageJ.

***RT-qPCR***

RNA was isolated using Universal RNA Purification Kit (EurX) and the reverse transcription was performed with AMV Reverse Transcriptase (EurX) according to manufacturer’s protocol. For qPCR, iTaq™ universal SYBR® Green Supermix (Bio-Rad) was used, and reactions were performed using Light Cycler 480 instrument (Roche). Sequences of primers used in the study: B2M (FP 5'-TGGAGGCTATCCAGCGTACT-3’, RP 5’-CGGATGGATGAAACCCAGAC-3’), β-actin (FP 5'-CATCCTCACCCTGAAGTACC-3', RP 5'-AGCCTGGATAGCAACGTACAT-3'), BCL2A1 (FP 5'-CAGAAGATGACAGACTGTGAA-3', RP 5'-TCCAAGCATGACTTCAGATTC-3'), CCL5 (FP 5'-GACACCACACCCTGCTGCT-3', RP 5'-TACTCCTTGATGTGGGCACG-3'), CXCL8 (FP 5'-CTGGCCGTGGCTCTCTTG-3', RP 5'-CCTTGGCAAAACTGCACCTT-3'), IL1B (FP 5'-ACGAATCTCCGACCACCACT-3', RP 5'-CCATGGCCACAACAACTGAC-3'), MLK4 (FP 5'-CATGAGGAGGCCTTCGTG-3’, RP 5’-CGCCAACCCAAAATCTGTAA-3’), NFKBIA (FP 5’-GAGTCAGAGTTCACGGAGTTC-3’, RP 5’-CATGTTCTTTCAGCCCCTTTG-3’), NFKBIZ (FP 5'-ATGGTGACACGTTCCTTCATA-3', RP 5'-CTGCACAATGAGATGCTGATT-3'), RELB, (FP 5'-CGTGCATGCTTCGGTCTGG-3', RP 5'-CTCCAATTCATCTGTGCTCC-3'), RPL29 (FP 5’-CAGCTCAGGCTCCCAAAC-3’, RP 5’-GCACCAGTCCTTCTGTCCTC-3’), TRAF1 (FP 5'-TCGATGGCACTTTCCTGTGGAAG-3', RP 5'-AGATGGGTTCTCTTTCCAGTGCC-3'). Primers were obtained from Genomed or Oligo.pl (Institute of Biochemistry and Biophysics Polish Academy of Science).

***Anchorage-dependent colony formation assay***

Cells were seeded at low seeding density in a 6-well plate format. For short-term colony formation assay, cells were transiently transfected the following day, using siRNA against MLK4 or non-targeting siRNA, and then left to grow for 5 days. For long-term colony formation assay cells were treated with 1 µg/ml doxycycline and left to grow for 2 weeks. Growth media was replaced every 2-3 days for doxycycline treatment. Colonies were then fixed with 4% PFA, then ice-cold methanol and stained with 0.5% crystal violet (Sigma) solution prepared in 25% methanol. Wells were thoroughly washed and air-dried. For quantification, 2 ml of 10% acetic acid was added to each well, incubated for 20 min with shaking and absorbance values were read at 590 nm.

***Cytotoxic/cytostatic and proliferation assays***

HCC1806 and HCC1599 doxycycline-inducible cell lines were seeded into 96-well plates and 1 µg/ml doxycycline was added into the medium the following day. After 6 days MTT Kit (Sigma) was used according to manufacturer’s instructions. Proliferation assay was performed in HCC1806 transiently transfected using MLK4-targeting or control siRNA. 72 hours following the transfection, cells were assayed for EdU incorporation (Click-iT™ EdU Microplate Assay, Thermo Fisher Scientific) according to manufacturer’s instructions.

***Cell cycle analysis***

HCC1806 cells were transiently transfected on 6-well plates using DharmaFECT1 (Dharmacon). After 72 hours cells were trypsinised and fixed in 70% ethanol for at least 24 hours. For flow cytometry, cells were treated with RNAse A (100 μg/ml) and stained with propidium iodide (40 μg/ml). Analysis was performed with LSR II Fortessa (BD).

***Single cell migration***

HCC1806 cells were treated with 1 μg/ml doxycycline. After 72 hours cell were seeded at low density. After cells attached, single cell motility was monitored for 20 hours using Iprasense Cytonote. Pictures of cells were taken every 20 min. Analysis of single cell motility was performed using ImageJ. Investigators were blinded when assessing the outcome of the assay.

***Phospho-kinase array***

MLK4 knock-down was induced by treatment with doxycycline for 72 hours. Then cells were lysed and Human Phospho-MAPK Array Kit (ARY002B) from R&D Systems was used according to manufacturer’s instructions.

***Evaluation of NF-κB p65 activity by DNA binding assay***

NF-κB p65 DNA binding activity was measured using ELISA-based colorimetric assay (TransAM, Active Motif). 20 µg of protein extracts were added per well and the signals were developed according to the manufacturer's instructions using microplate reader (Biotek).

***Gene Set Enrichment Analysis***

GSEA (9) was performed using GSEA version 3.0 from the Broad Institute at MIT. Two datasets were analyzed by GSEA: The Cancer Genome Atlas (TCGA) RNA-Seq dataset including 817 (82 TNBC) breast cancer samples and GSE76275 dataset from RNA profiling using Affymetrix HG-U133 Plus 2,0 GeneChips array including 265 breast cancer samples (198 TNBC and 67 non-TNBC). For GSEA, the list’s ranking metric was calculated on the Pearson correlation coefficient between MLK4 expression levels and all the genes identified in the analyzed cohorts. The “c2.all.v6.1.symbols.gmt” gene sets were downloaded from the Broad Institute GSEA website ([http://software.broadinstitute.org/gsea/index.jsp)](http://software.broadinstitute.org/gsea/index.jsp)a) and used for the analysis. To calculate *P*-values 1000 permutations were used and permutation type was set to phenotype. The expression level of MLK4 (KIAA1804; 228565_at) was used as phenotype label, and “Metric for ranking genes” was set to Pearson Correlation. The other basic and advanced fields were set to default.

***Immunohistochemistry***

A total of 129 samples of breast carcinoma from patients who underwent radical or partial mastectomy between 2008-2015 at the University Clinical Centre in Gdansk were examined (Table S1). This included 7 lobular carcinomas (LC), 5 mucinous carcinomas (MC), 4 metaplastic carcinomas (MetC), 4 invasive micropapillary carcinoma (MPC), 1 atypical medullary carcinoma (MedC), and 108 invasive carcinomas of no special type (NST). Based on the receptor profile, 46 of these cancers were diagnosed as triple-negative, 31 as luminal B-like, 26 as luminal A-like and 26 as HER2 positive according to the definitions used by St Gallen International Expert Consensus (10). Clinical and pathological information were collected from pathologic reports. The study was approved by the Independent Bioethics Commission for Research of Medical University of Gdansk. Immunohistochemical staining for MLK4 was performed manually on 4 μm-thick freshly cut sections of formalin-fixed, paraffin-embedded blocks of tumor tissues. For each patient, a representative block was chosen based on the evaluation of hematoxylin and eosin-stained sections. The pretreatment was performed using heat-induced antigen retrieval in pH 6,1 (Target Retrieval Solution S1699 Dako) with subsequent overnight incubation at 4°C with polyclonal rabbit anti-human MLK4 antibody (Bethyl Laboratories) diluted 1:50 with Antibody Diluent (K8006, Dako Co.). Antibody incubation was followed by the standard signal amplification, with rabbit LINKER (K8009) at room temperature for 15 min, HRP-conjugated EnVision™ FLEX+ (K8002) at room temperature for 30 min, DAB reaction for 10 min and counterstaining with hematoxylin for 6 min. In each sample the staining was evaluated using the H-score system (11) by two pathologists who were blinded to the patho-clinical data at the point of assessment. To validate the MLK4 antibody we have used the positive control from healthy cerebellum and breast tissues, as well as negative reagent control. The positive control was chosen based on information provided in the Human Protein Atlas (<https://www.proteinatlas.org/ENSG00000143674-MAP3K21/tissue>). All samples contained an internal control (residual healthy breast tissue), and the staining was repeated in the cases which showed weak or moderate instead of strong reaction in the control. After the repeated staining two cases with questionable staining quality were removed from the study, with no influence on the overall results.

**Supplementary Figure Legends**

**Supplementary Figure 1. MLK4 is upregulated in invasive breast carcinoma.** **a** Amplification and mRNA upregulation of MLK1-4 in invasive breast carcinoma (n=818) from TCGA dataset. Graphic illustrations taken from cBio Portal. **b-d** Volcano plots illustrating changes in mRNA expression (log2 fold change versus -log10 of *P*-value) between TNBC samples and HER2+ or ER/PR+ samples. Analysis performed for three independent data sets indicate that MLK4 is usually among top 1% of genes with the highest probability of being differentially expressed.


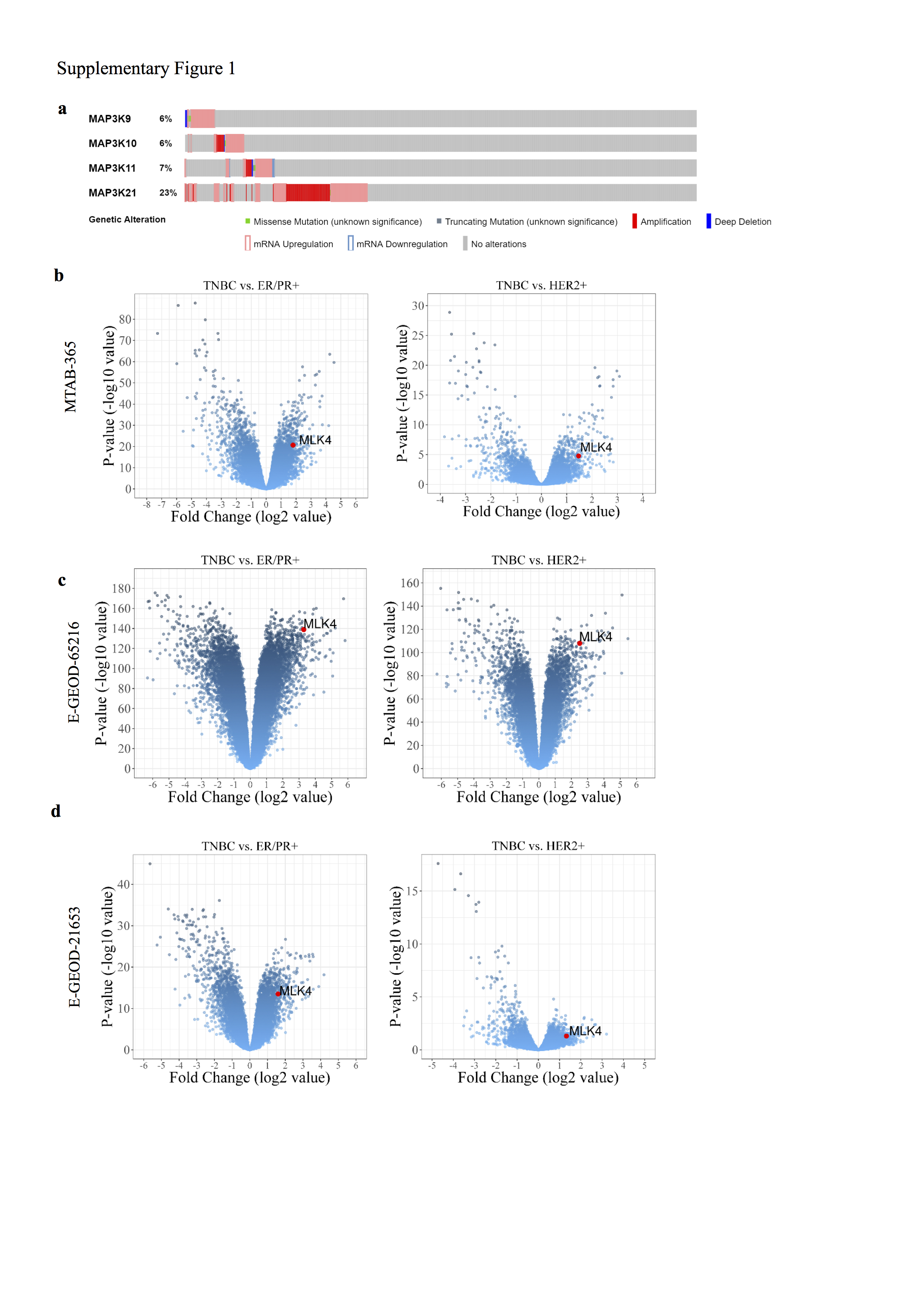


**Supplementary Figure 2. Probability of survival in breast cancer patients expressing high or low MLK1-4 levels. a-b**, Probability of recurrence-free survival in all breast cancer patients (**a**) or TNBC (**b**) patients expressing high or low MLK4 levels assessed using KMplotter, with auto-selected best cutoff. Graphic illustrations taken from Kmplot.com. **c** Probability of overall survival in breast cancer patients expressing high or low MLK1-3 levels assessed using KMplotter, with auto-selected best cutoff. Graphic illustrations taken from Kmplot.com.


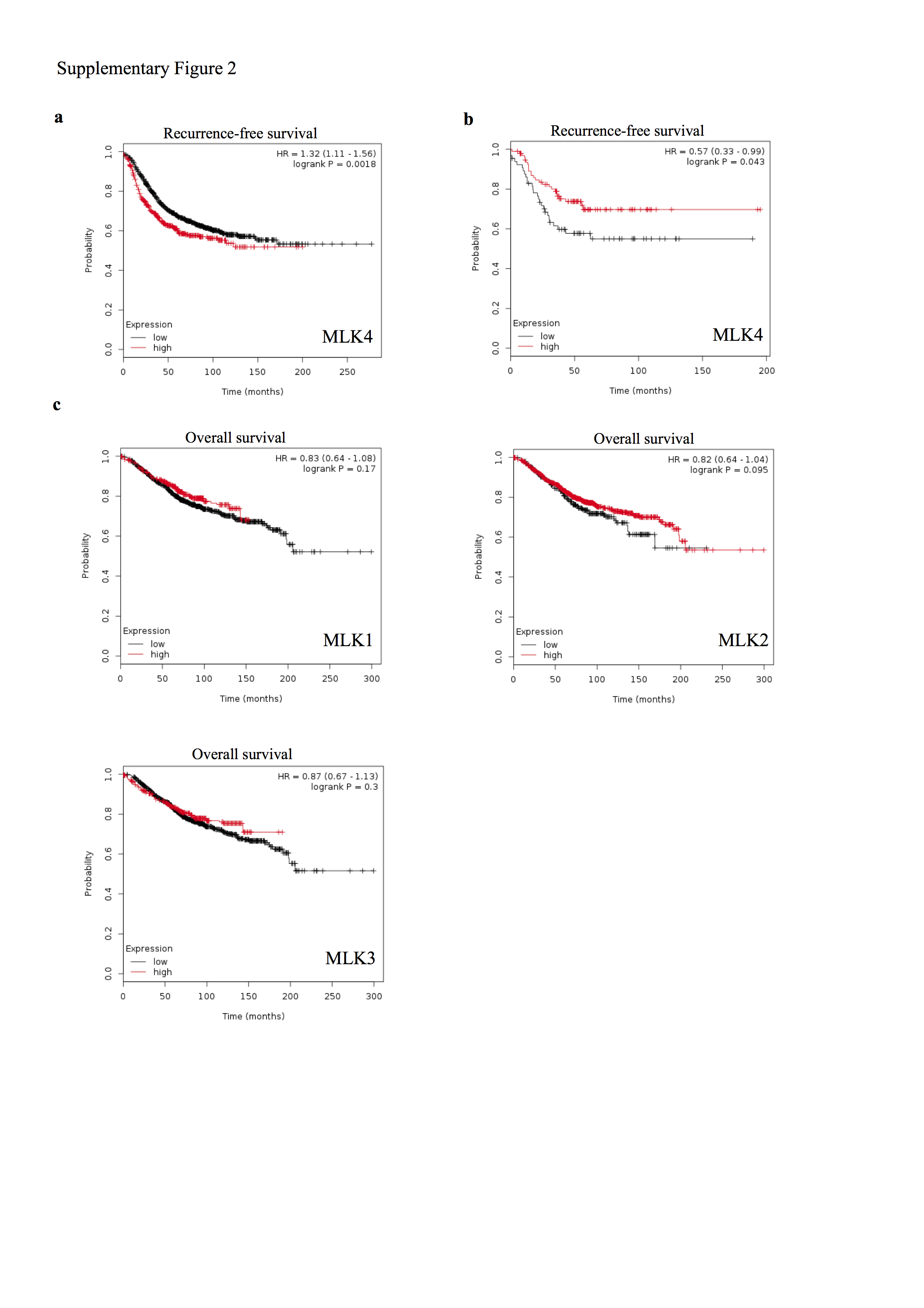


**Supplementary Figure 3.** **MLK4 protein abundance across breast cancer cell lines and MCF10A.** Lysates from all cell lines were prepared and analyzed by western blotting. The figure shows representative image for 3 independent experiments that were used for quantification analysis shown in Fig. 2a.

**
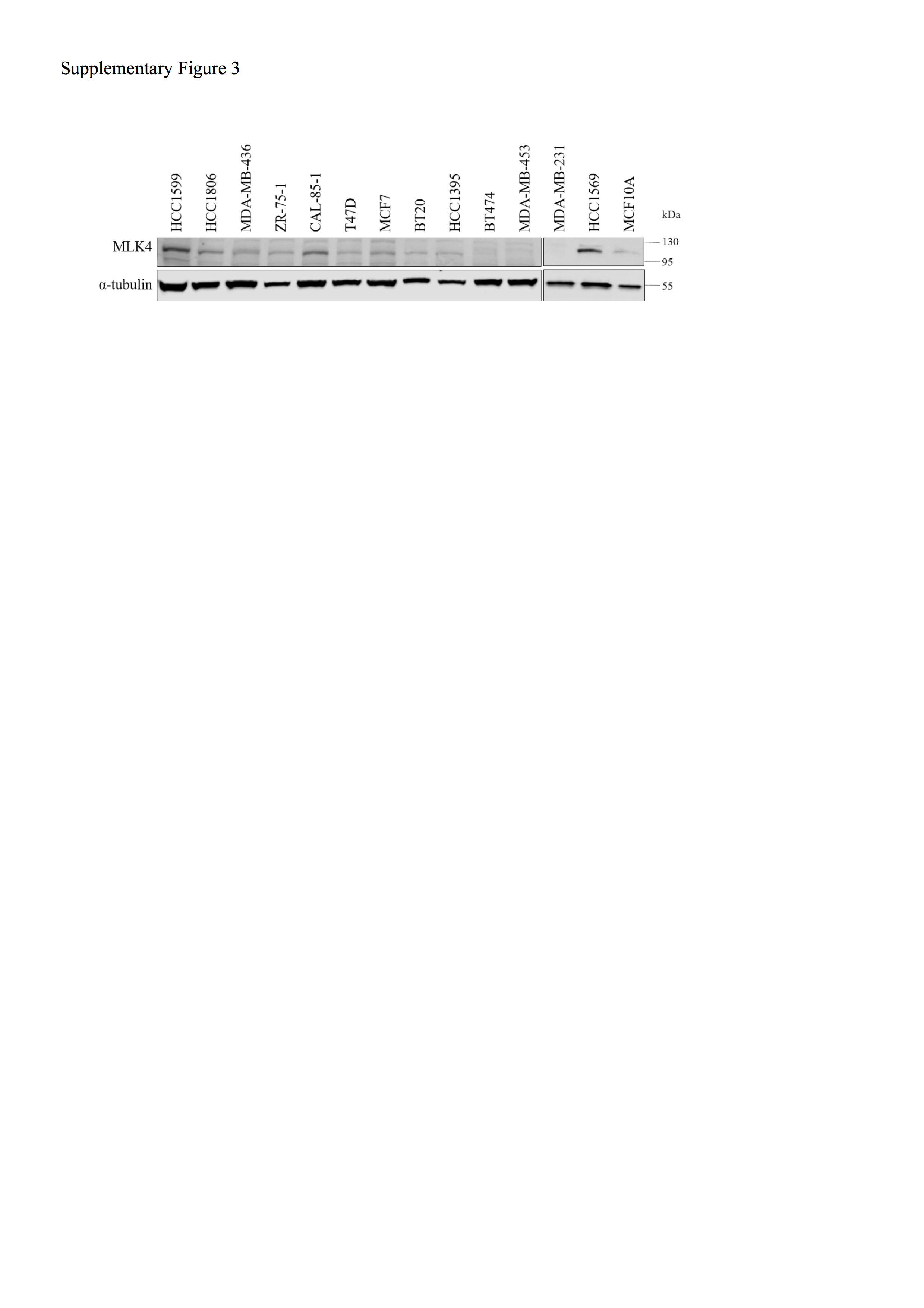
**

**Supplementary Figure 4**. **Cell proliferation is not affected by doxycycline treatment of parental HCC1806 cells.** Five thousands of HCC1806 cells were seeded and were grown with or without 1 μg/ml doxycycline for 10 days. At day 6, 8 and 10 cells were counted using Bio-Rad Cell Counter TC-20. Error bars indicate ±SEM from three independent experiments (n=3). Statistical comparison of values was performed using the unpaired two-tailed *t*-test. Results are not statistically significant. Representative immunoblots showing the level of MLK4 after doxycycline treatment (no changes in parental HCC1806 cells) are on the right.


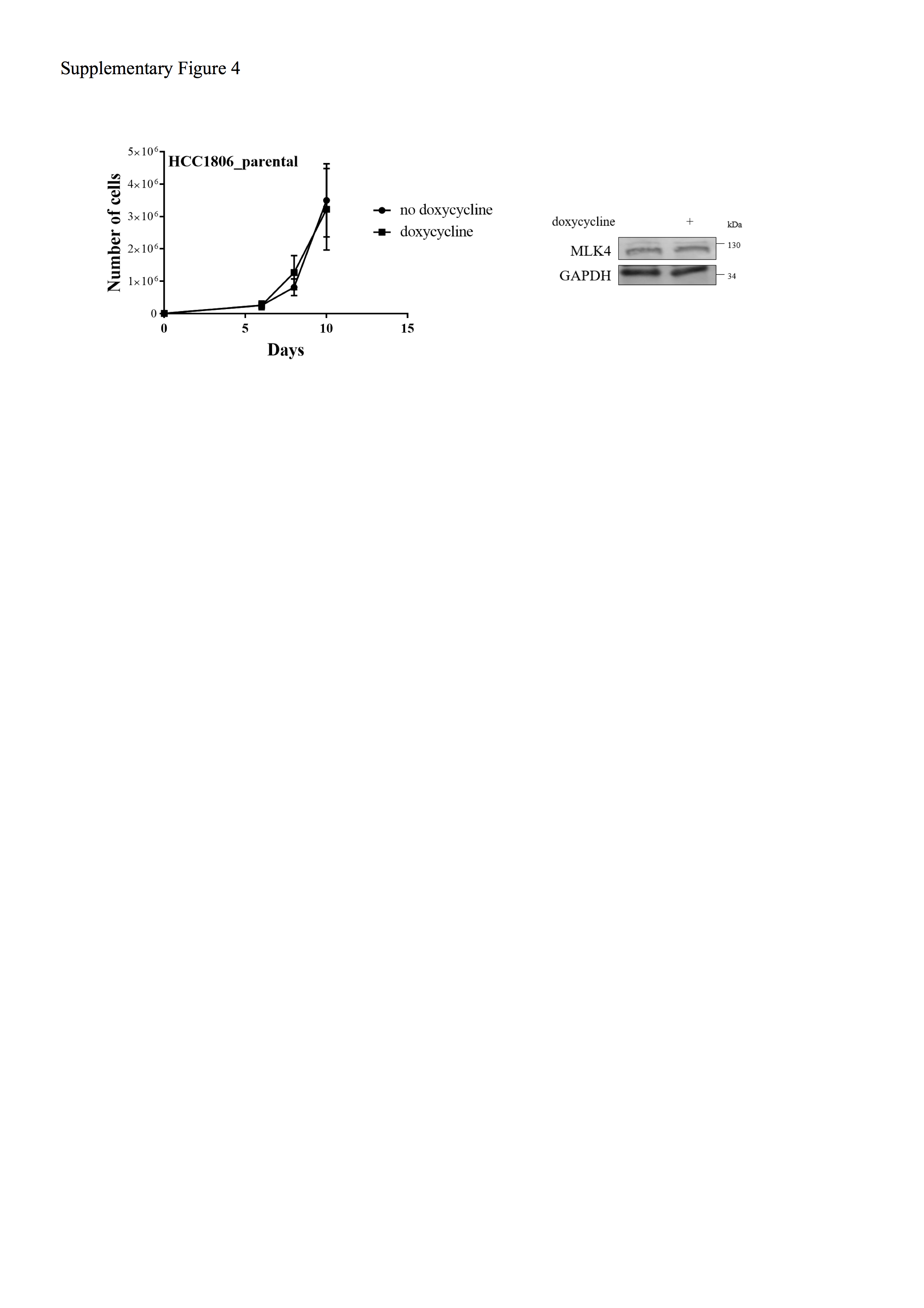


**Supplementary Figure 5. MLK4 depletion leads to cell cycle arrest of TNBC cells with high expression of MLK4.** **a** Parental HCC1806 cells were transfected with siRNA against MLK4 or control siRNA. 72 hours following the transfection, cells were lysed and analyzed by western blotting. **b** Parental HCC1806 cells were transfected with siRNA against MLK4 or control siRNA. 72 hours following the transfection, cells were harvested and fixed in 70% ethanol. Cells then were treated with RNAse A and stained with propidium iodiode. Representative flow cytometry traces are shown (P5 – Sub G1, P6 – G0/G1, P7 – S, P8 – G2/M).

**
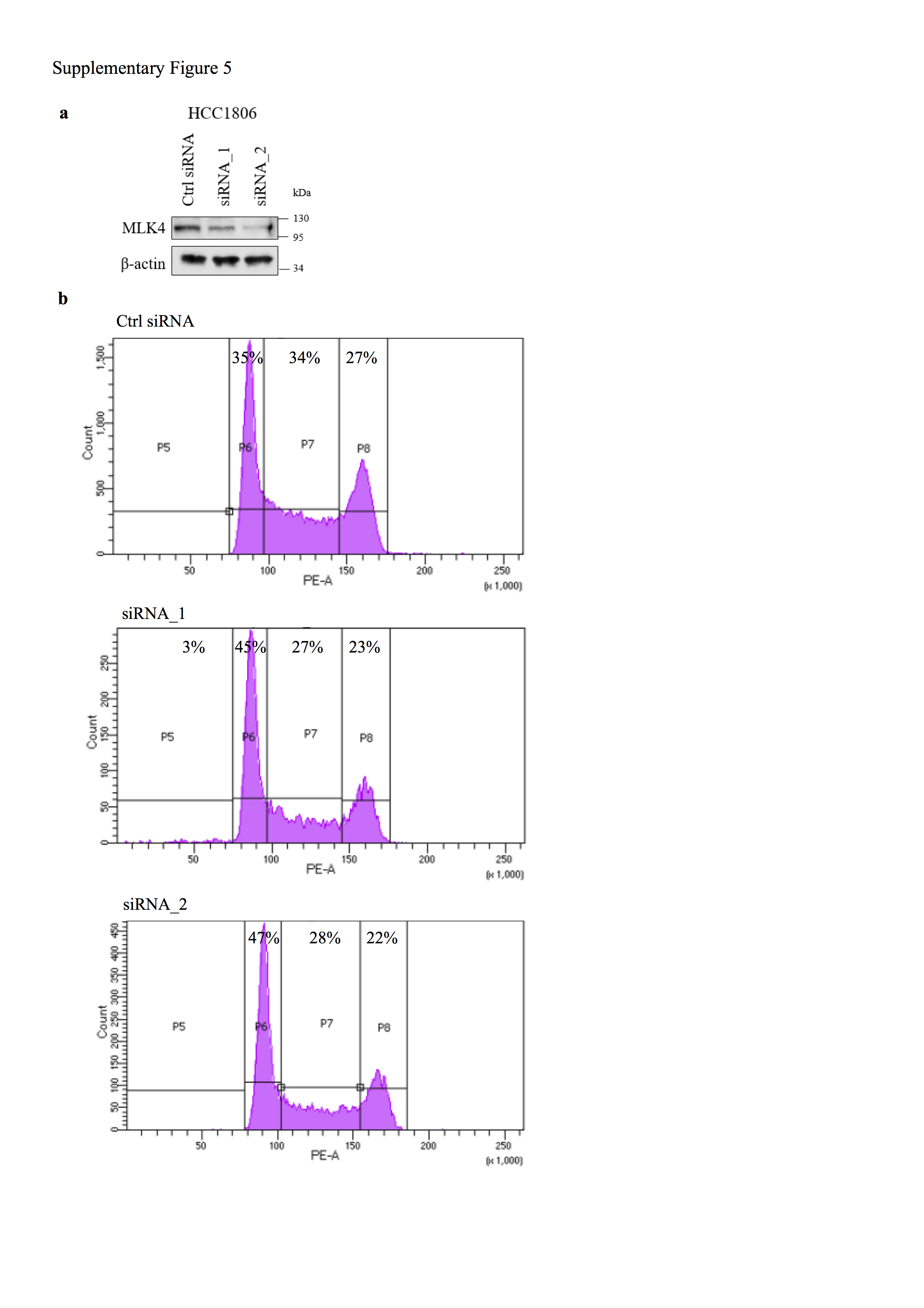
**

**Supplementary Figure 6. Overexpression of MLK4 in BT474 leads to increased migratory potential.** BT474 cells with doxycycline-inducible overexpression of MLK4 were generated. Then cells were treated with 1 μg/ml doxycycline for at least 72 hours, then cells were serum-starved and seeded on transwell inserts. The cells were allowed to migrate for no longer than 24 hours. Five pictures of every condition were taken. Analysis was performed using ImageJ. Error bars indicate ±SEM from three independent experiments (n=15). Statistical analysis was done using unpaired two-tailed *t*-test. * *P*<0,05. On the right, representative immunoblots showing the level of MLK4 overexpression.


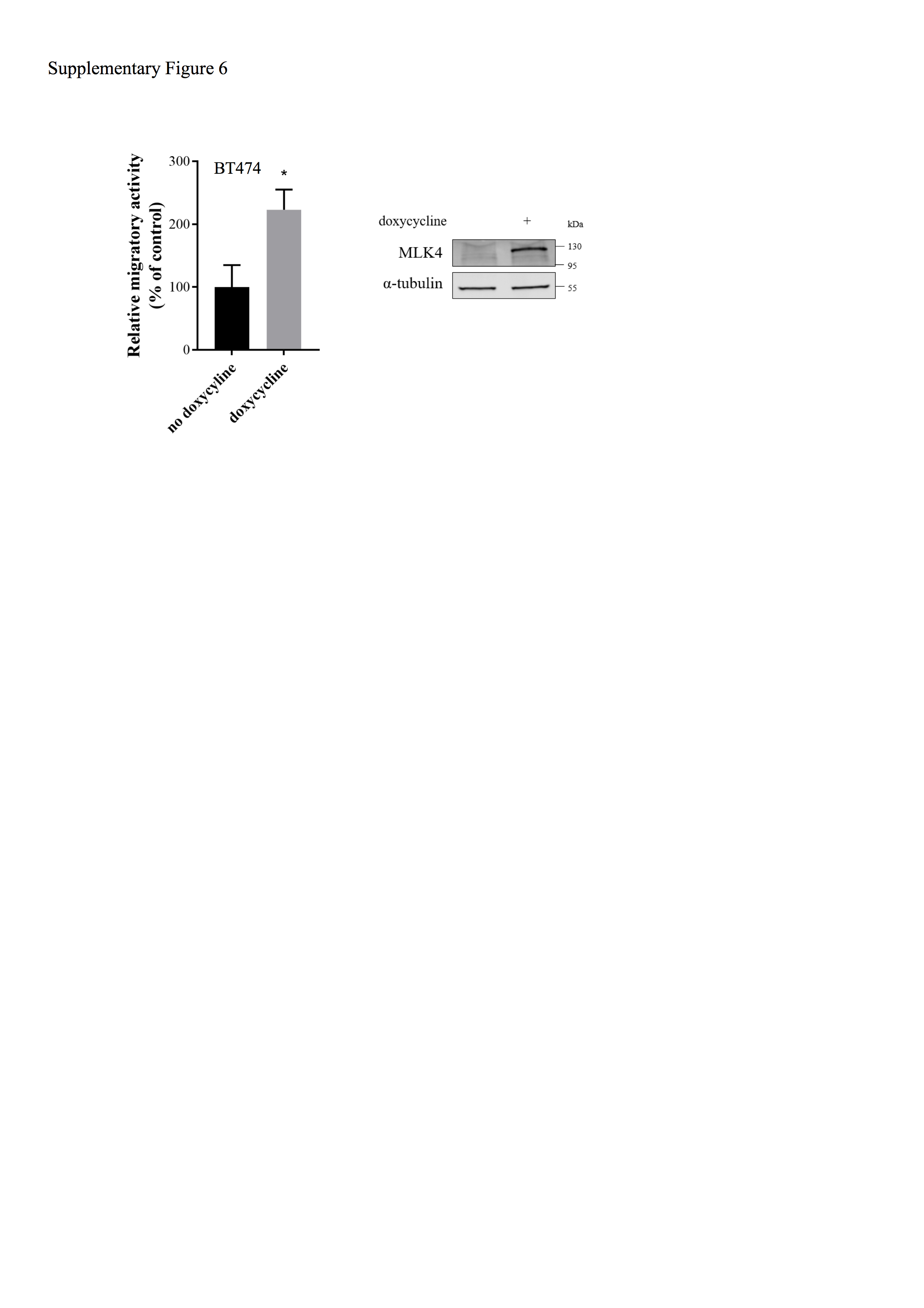


**Supplementary Figure 7**. **Migration, invasion and 3D growth is not affected by doxycycline treatment of parental HCC1806 cells. a** and **b** Parental HCC1806 cells were treated with 1 μg/ml doxycycline for at least 72 hours, then cells were serum-starved and seeded on transwell inserts uncoated (**a**) or coated with Matrigel (**b**). The cells were allowed to migrate for no longer than 24 hours. Cells that migrated through the inserts were stained with crystal violet and five pictures of every condition were taken. Analysis was performed using ImageJ. Error bars indicate ±SEM from three independent experiments (n=15). Statistical analysis was done using unpaired two-tailed *t*-test. Results are not statistically significant. Below, representative immunoblots showing the level of MLK4 after doxycycline treatment (no changes in parental HCC1806 cells). **c** HCC1806 cells were treated with 1 μg/ml doxycycline for at least 72 hours and then were subjected to wound healing assay. Four pictures were taken at each condition and quantification was performed using ImageJ. Error bars indicate ±SEM from three independent experiments (n=12). Statistical analysis was done using unpaired two-tailed *t*-test. Results are not statistically significant. On the right, representative immunoblots showing the level of MLK4 after doxycycline treatment (no changes in parental HCC1806 cells). **d** HCC1806 cells were seeded on plates coated with Matrigel. Cells were grown for 10 days with or without 1 μg/ml doxycycline. Pictures were taken and the number of spheroids was quantified in ImageJ. Error bars indicate ±SEM from three independent experiments. Statistical comparison of values was performed using the unpaired two-tailed *t*-test. Results are not statistically significant. **e** Parental HCC1806 cells were injected into mammary fat pads of RAG2^-/-^ mice. Doxycycline administration started one day after the injection. Tumors were measured twice a week. Error bars indicate ±SEM (n=8 for both groups). Statistical comparison of values was performed using the unpaired two-tailed *t*-test. Results are not statistically significant.


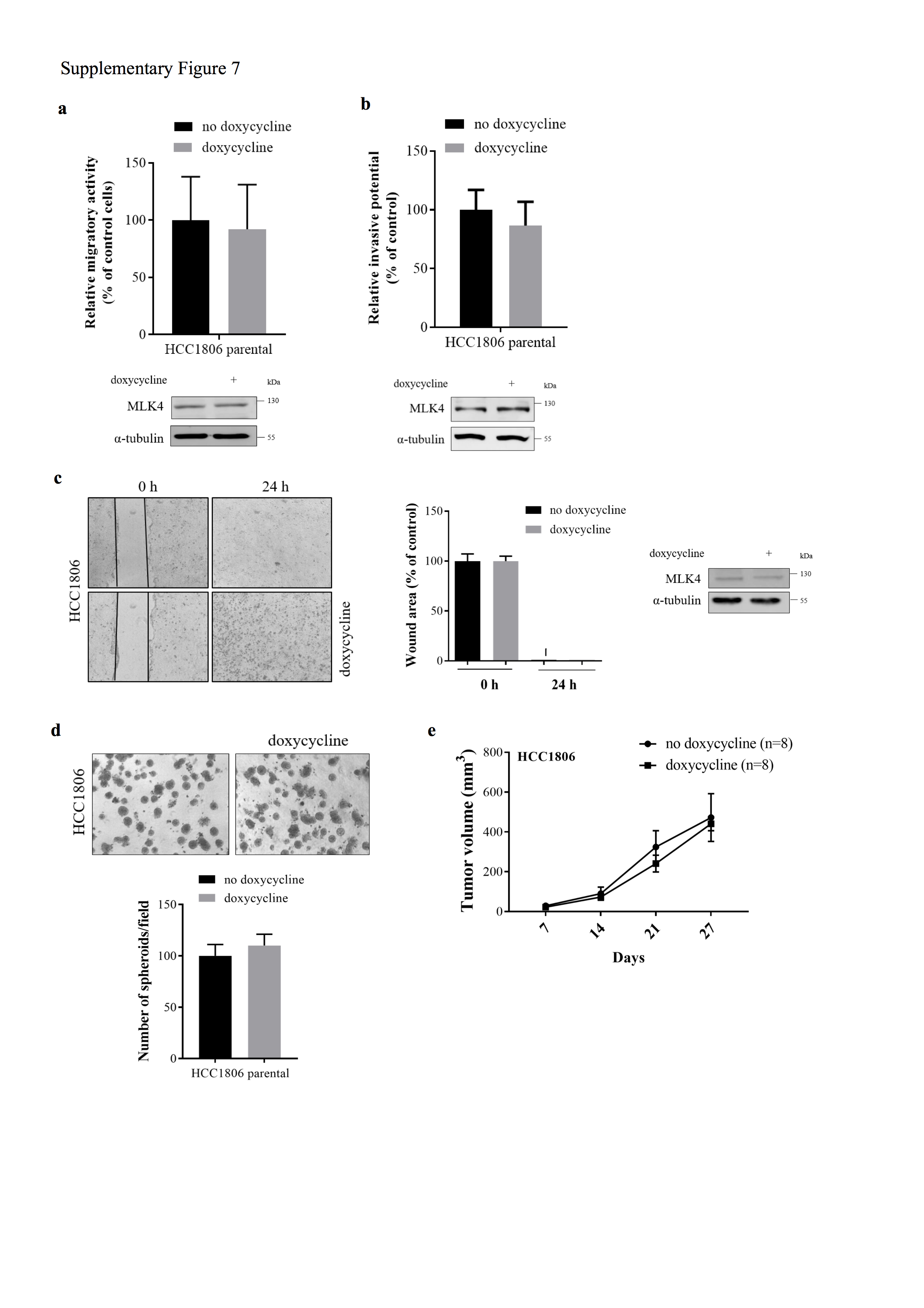


**Supplementary Figure 8.** **MAPK pathway activity is not affected by MLK4 knock-down in HCC1806 cells.** **a** HCC1806_sh6 were treated with 1 μg/ml doxycycline for 72 hours. Then cells were lysed and subjected to Human Phospho-MAPK Array Kit. Representative immunoblots showing the level of MLK4 knock-down is shown below. **b** Quantification of p-ERK1/2 signals from three independent experiments was performed using ImageJ (n=3). Statistical comparison of values was performed using the unpaired two-tailed *t*-test. Results are not statistically significant.


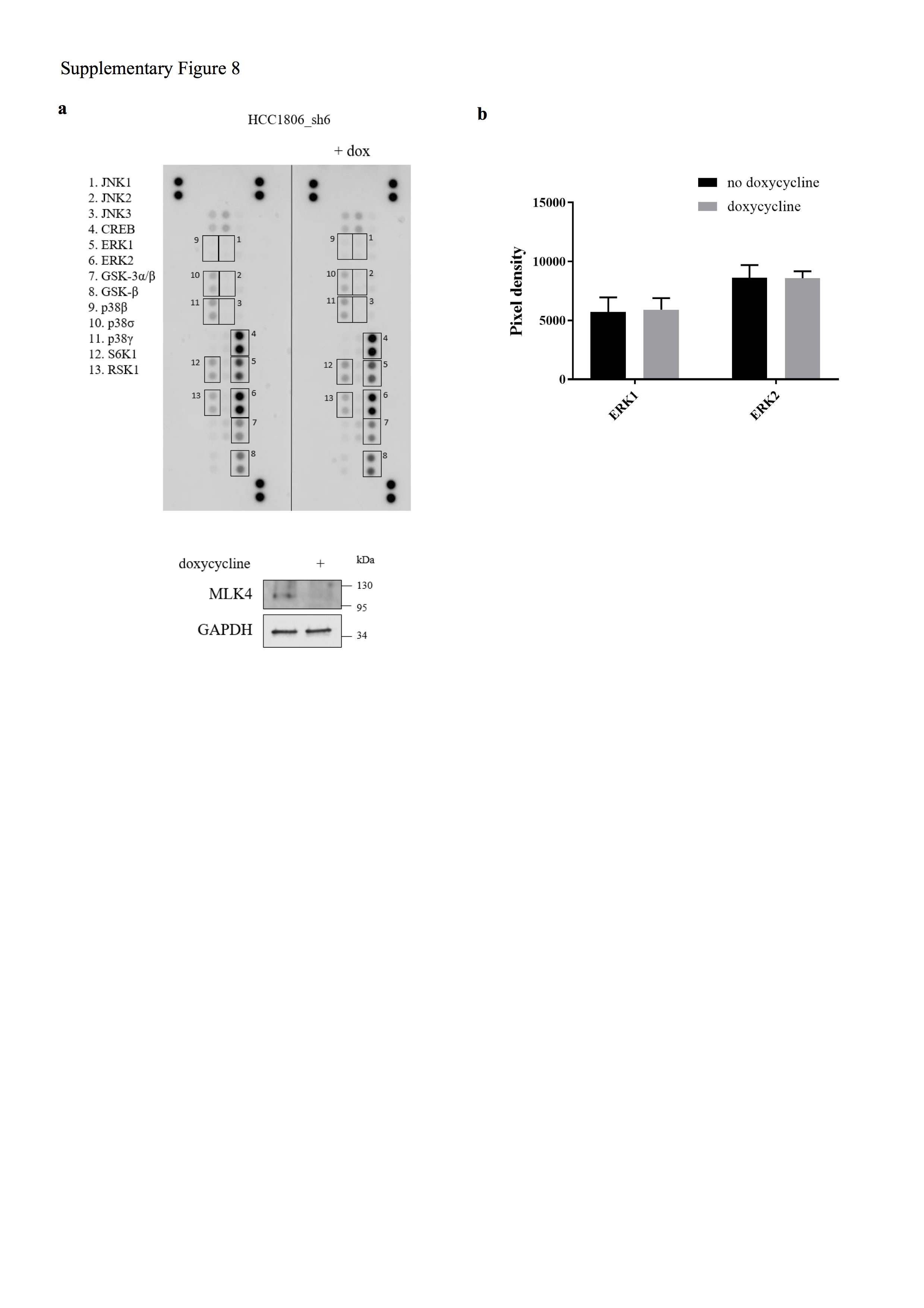


**Supplementary Figure 9. MLK4 knock-down leads to decreased phosphorylation of NF-κB in HCC1806 cells.** HCC1806_sh2 and HCC1806_sh6 cells were treated with 1 μg/ml doxycycline for 72 hours, then were stimulated with 20 ng/ml TNF-α for indicated time points. Whole cell lysates were analyzed by western blotting (**a**). Quantification analysis of immunoblots was performed using ImageJ (**b**). Error bars indicate ±SEM from four independent experiments (n=4). Statistical comparison of values was performed using the unpaired two-tailed *t*-test. *** *P*<0,001, ** *P*<0,01, * *P*<0,05.

**
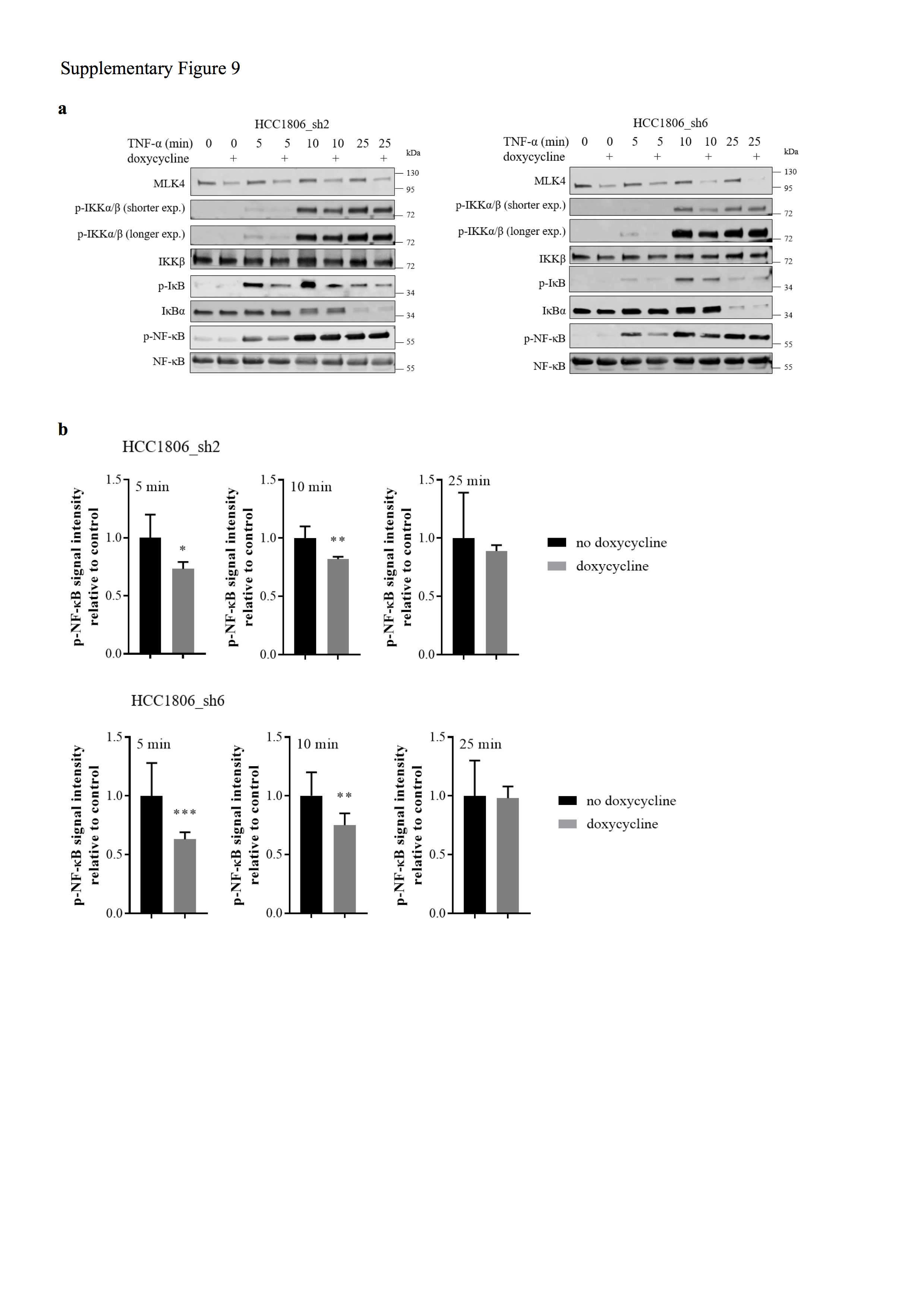
**

**Supplementary Figure 10. Activity of NF-κB pathway is not affected by doxycycline treatment of parental HCC1806 cells.** **a** Parental HCC1806 cells were treated with 1 μg/ml doxycycline for 72 hours, then were stimulated with 20 ng/ml TNF-α for indicated time. Whole cell lysates were analyzed by western blotting. **b** HCC1806 cells were treated with 1 μg/ml doxycycline for 72 hours, and then were stimulated with 20 ng/ml TNF-α for 1h, lysed and subjected to TransAM DNA binding assay using NF-κB p65-specific antibody. Error bars indicate ±SEM from three independent experiments (n=3). Statistical comparison of values was performed using the unpaired two-tailed *t*-test. Results are not statistically significant.


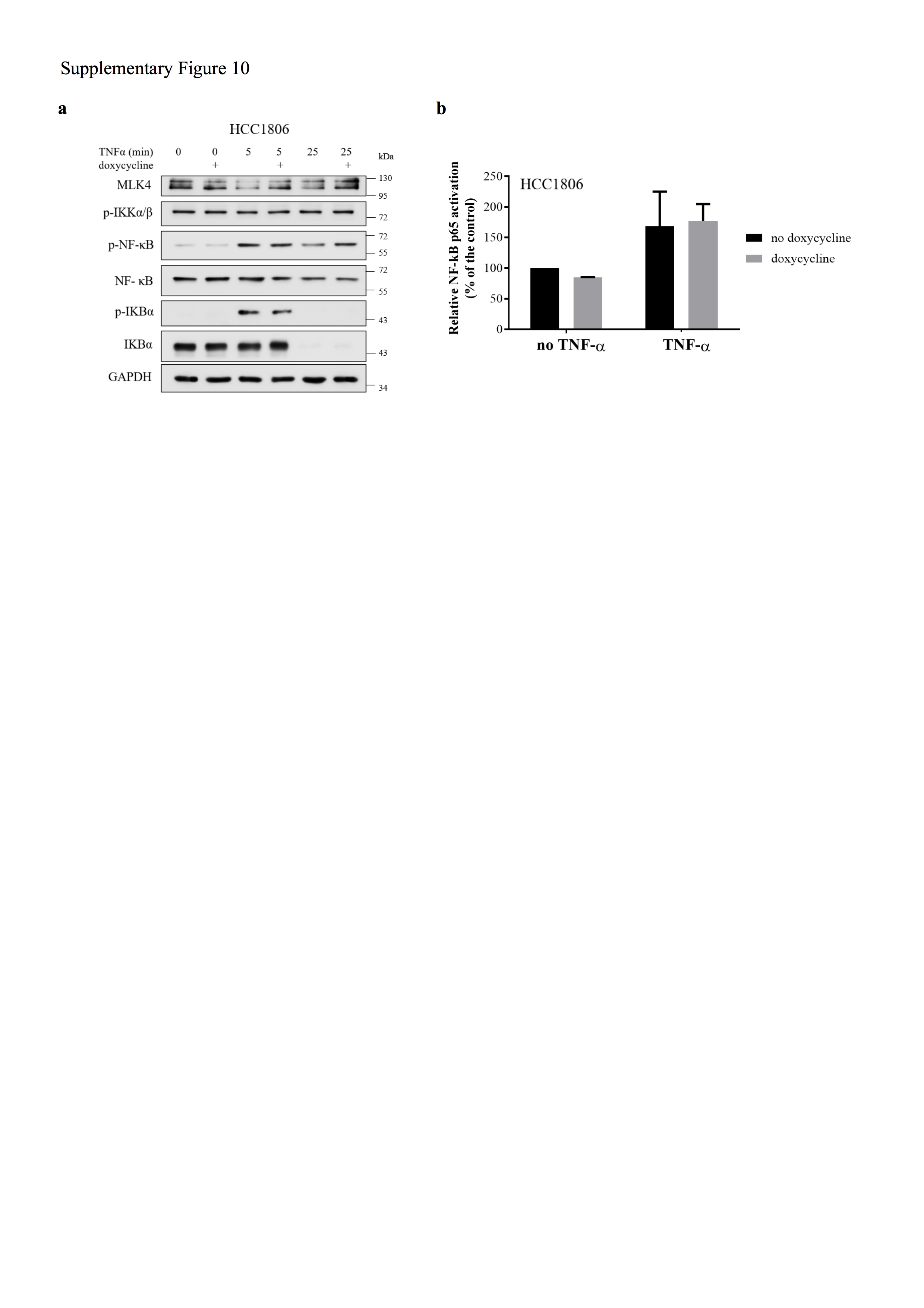


**Supplementary Figure 11. MLK4 depletion leads to downregulation of NF-κB target genes in TNBC cells.** HCC1806_sh6 cells were treated with 1 μg/ml doxycycline for 72 hours, and then were stimulated with 20 ng/ml TNF-α for 6 hours before RNA isolation. NF-κB target genes mRNA abundance was determined using RT–qPCR, relative to beta-actin. Error bars indicate ±SEM from three independent experiments (n=3). RT-qPCR data were assessed using one-way ANOVA followed by Bonferroni's multiple comparison tests. **** *P*<0,0001, *** *P*<0,001, ** *P*<0,01, * *P*<0,05.


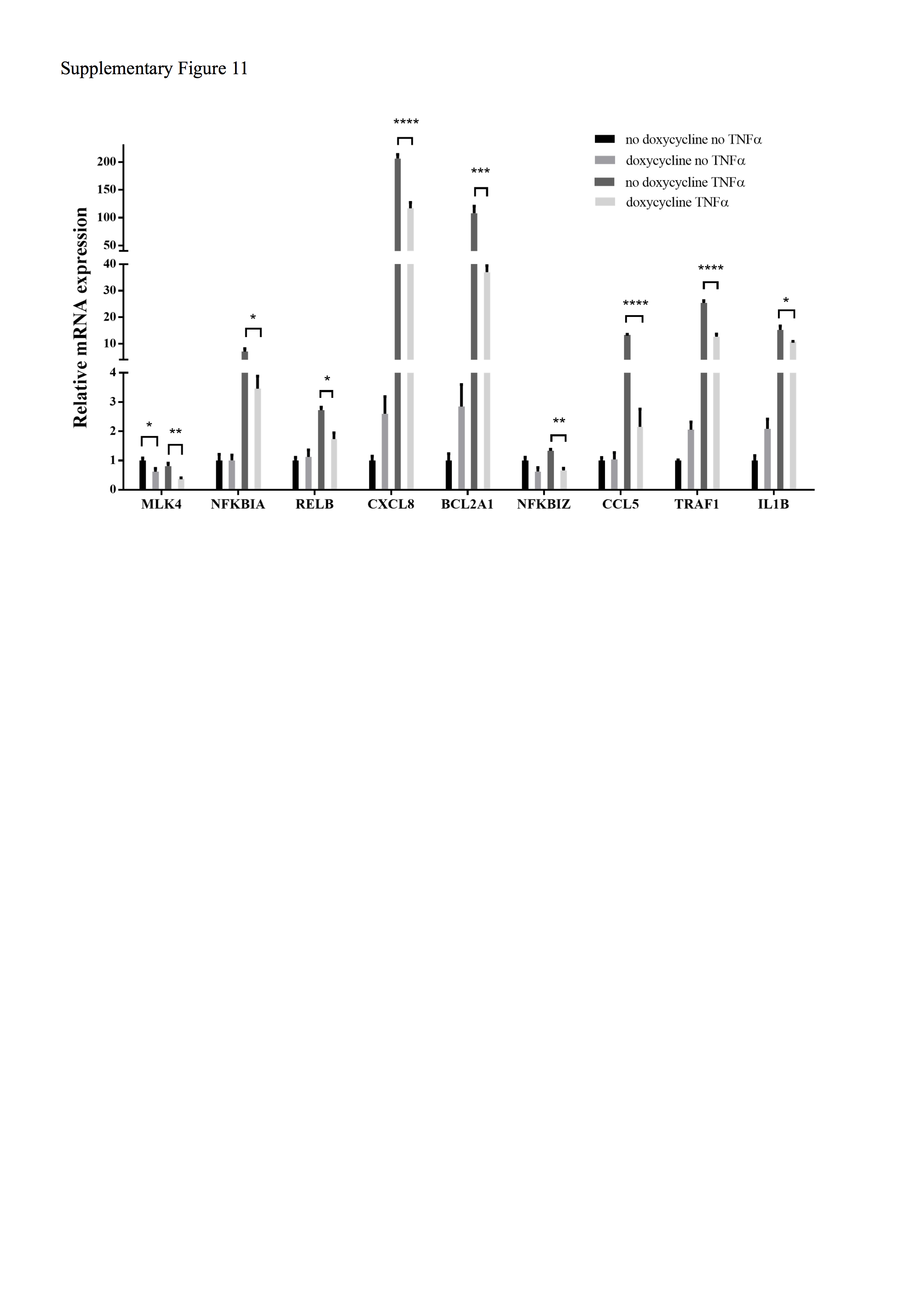


**Supplementary Figure 12. Overexpression of MLK4 in BT474 cell line leads to upregulation of mesenchymal markers and increased phosphorylation of NF-κB.** BT474 cells were treated with 1 μg/ml doxycycline for 72 hours. Whole cell lysates were analyzed by western blotting.


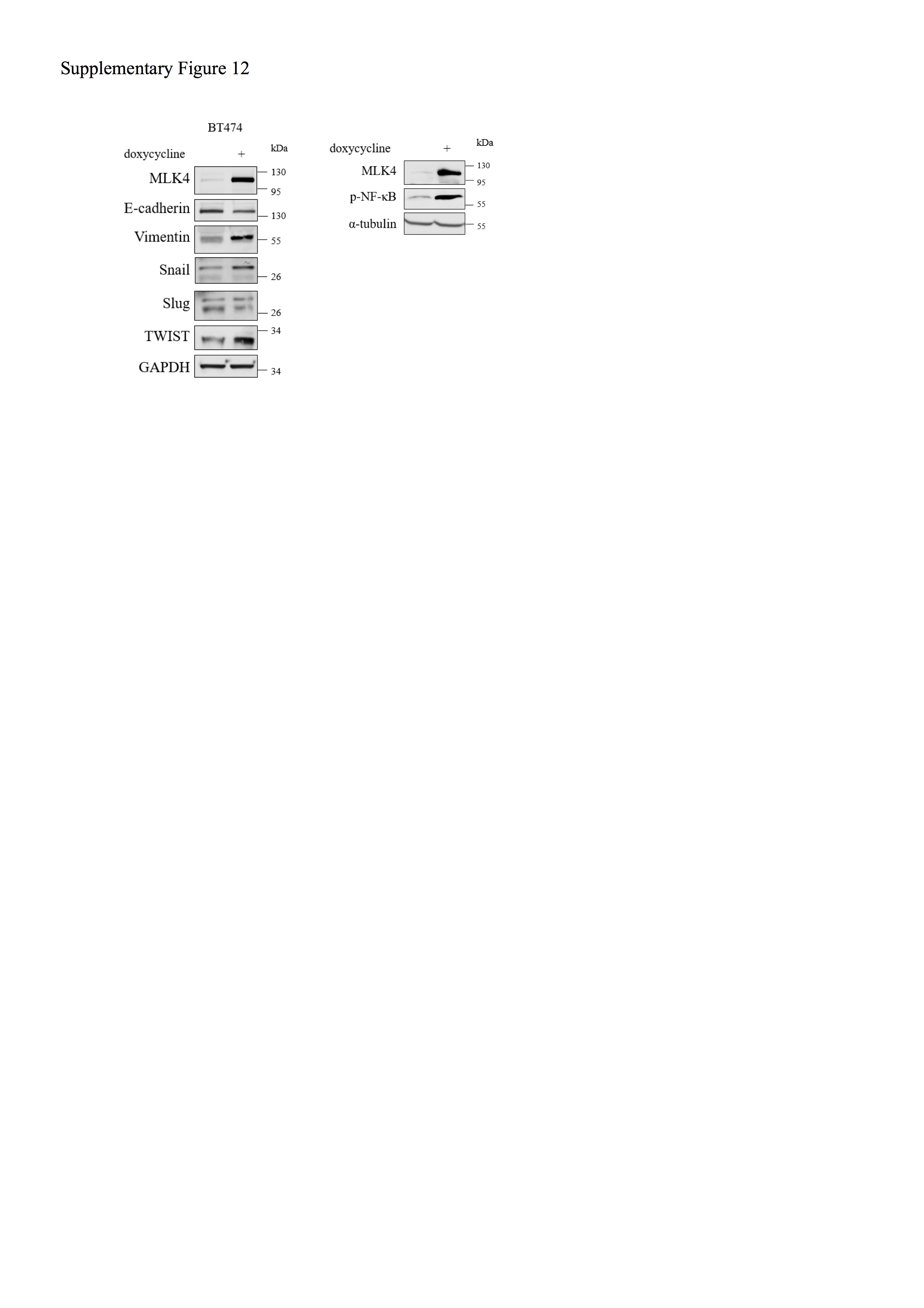


**Supplementary Figure 13. Gene set enrichment analysis (GSEA) shows a positive correlation between MLK4 mRNA levels and EMT-related genes expression in clinical specimens.** GSEA was performed on TCGA RNA-seq and GSE76275 datasets (12,13). Genes were ranked according to correlation with MLK4 expression. Significance was determined based on NES and FDR-q values.


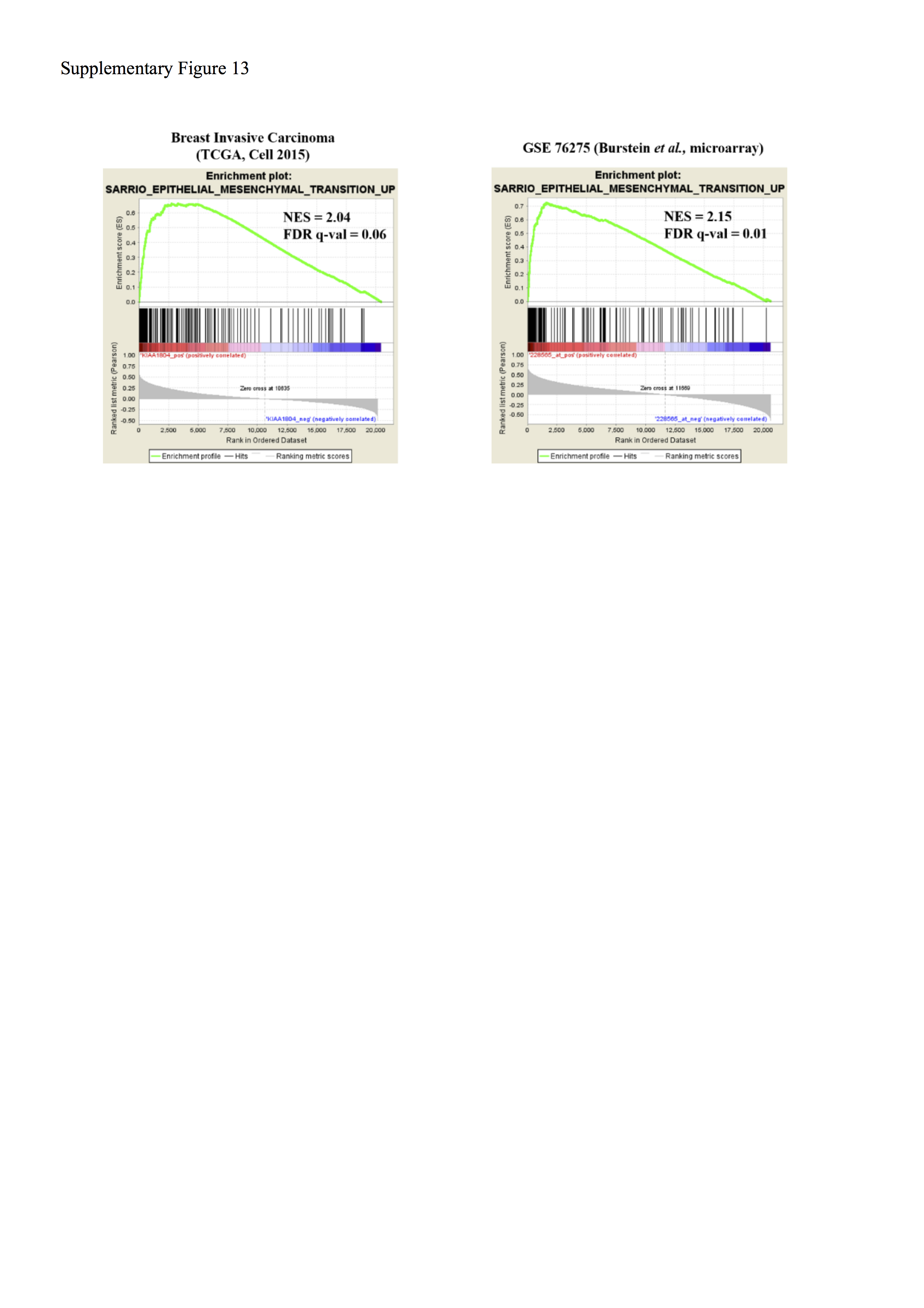


**Supplementary Figure 14. Blocking NF-κB pathway activity in parental HCC1806 cell line decreases anchorage-dependent colony formation and migration of cells. a** Parental HCC1806 cells were seeded at low density and were grown in the presence of NF-κB pathway inhibitor, BAY-11-7082, or DMSO control. After 10 days, cells were stained with crystal violet and results quantified by absorbance. Error bars indicate ±SEM from three independent experiments (n=3). Statistical comparison of values was performed using the unpaired two-tailed *t*-test. ** *P*<0,001. **b** Parental HCC1806 cells were seeded on transwell inserts. When cells attached to the bottom of inserts, the medium was changed and cells were treated with 10 µM BAY-11-7082 or DMSO control overnight. Cells that migrated through the inserts were stained with crystal violet. Five pictures of every condition were taken. Analysis was performed from three independent experiments using ImageJ (n=15). Statistical analysis was done using unpaired two-tailed *t*-test. ** *P*<0,001.


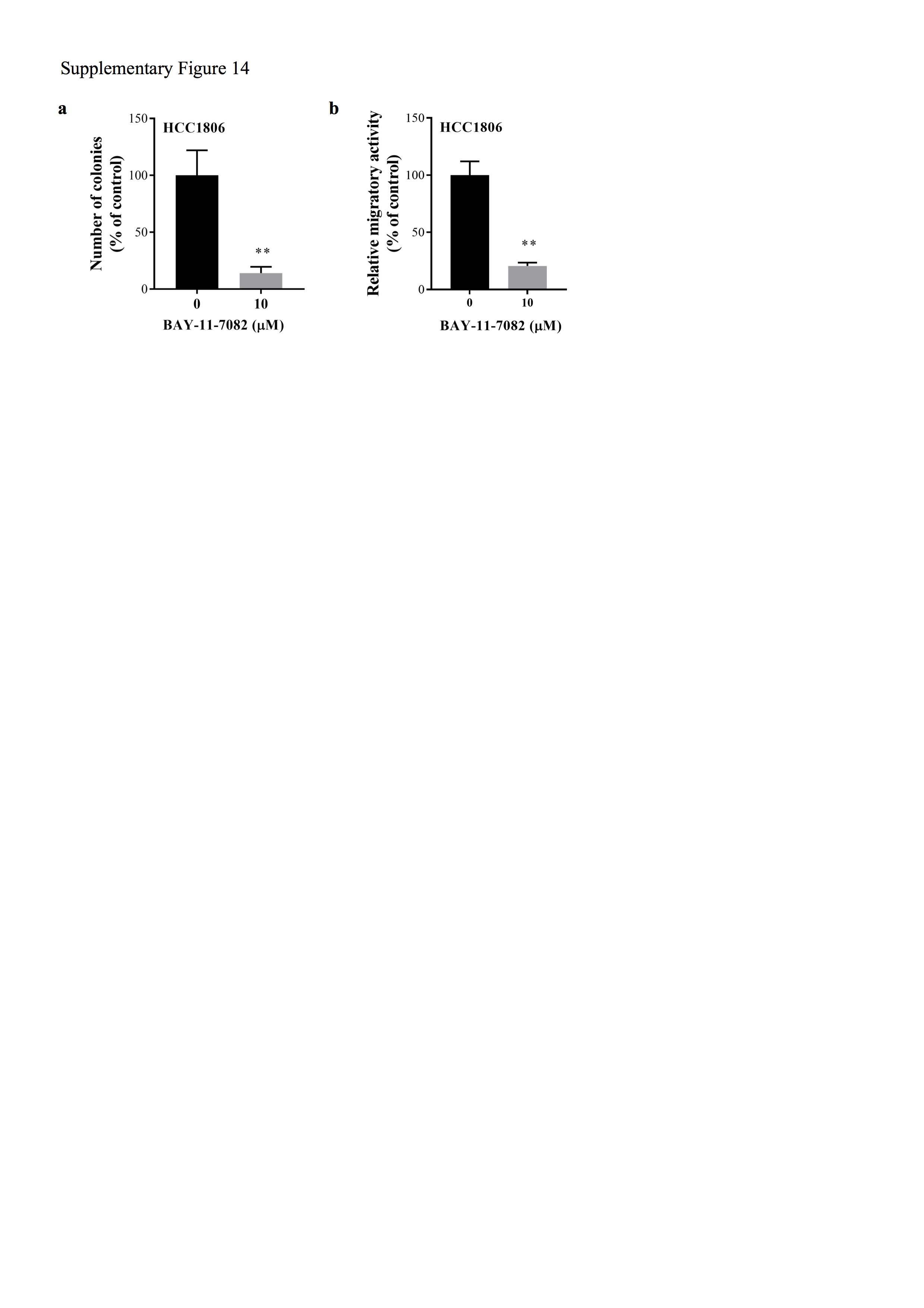


**Supplementary Figure 15.** Weak, moderate, and strong intensity of MLK4 staining in different cases of breast carcinoma. Scale bar 20 µm.

**
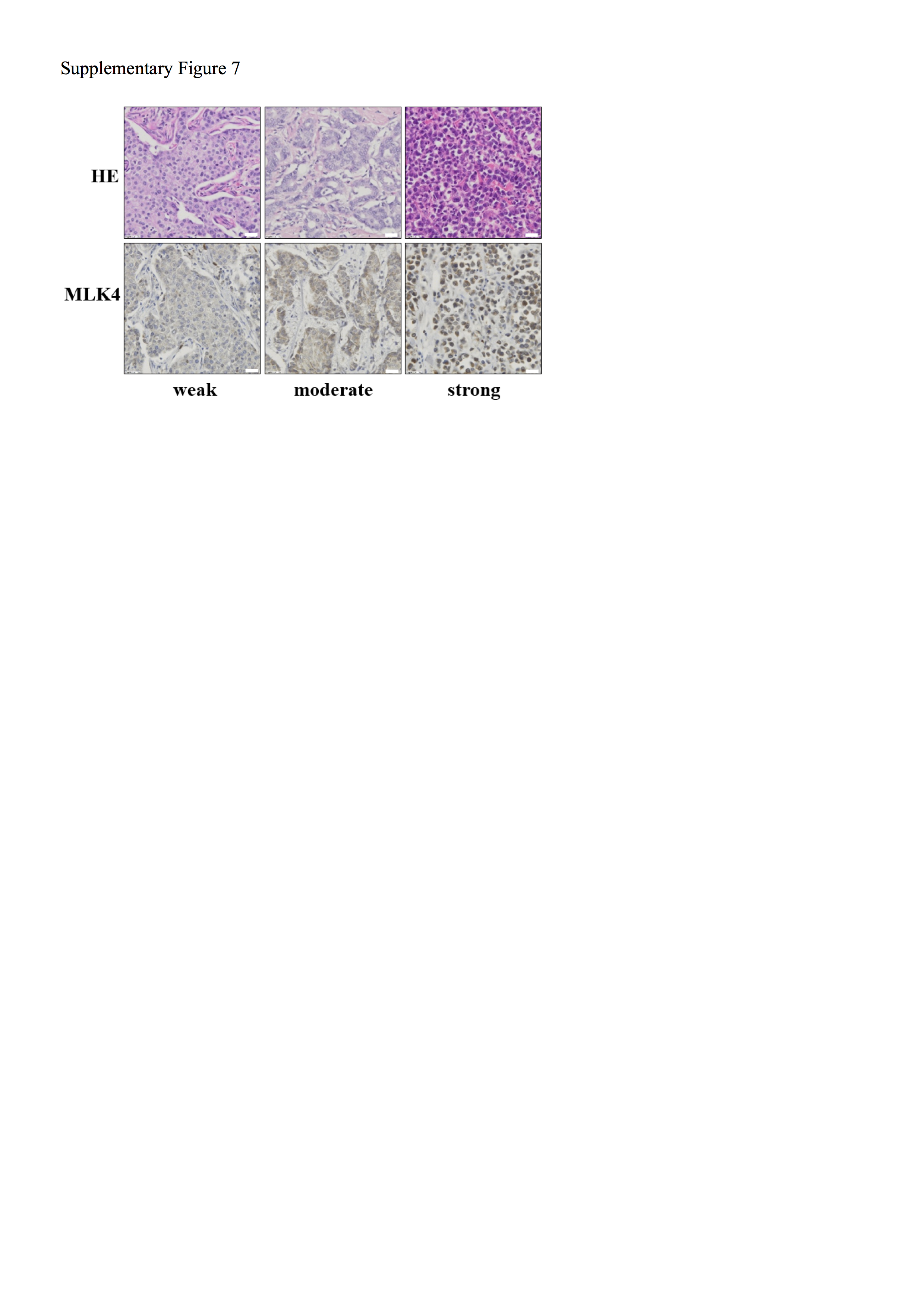
**

**Supplementary Table 1.** High MLK4 expression is associated with lymph nodes metastasis in TNBC patients. Characteristics of patients, histopathological diagnosis, BC molecular subtype, grade and information on occurrence of lymph node metastasis.

| **No** | **Age** | **Histopathological diagnosis**  **(D-ductal, L-lobular, M-mucinous, MI-micropapillary, MP-metaplasticum, NST-not otherwise specified, HC-histiocytoid, ME-atypical medullary)** | **Molecular subtype (A- luminal A; B - luminal B; H - HER2+ ; N-triple negative)** | **Grade** | **H score** | **N>0 (lymph node metastasis)** |
| --- | --- | --- | --- | --- | --- | --- |
| **1** | 58 | NST | A | 2 | 285 | 0 |
| **3** | 59 | NST | A | 2 | 140 | 0 |
| **4** | 58 | NST | A | 1 | 205 | 0 |
| **6** | 65 | NST | A | 2 | 130 | 1 |
| **7** | 56 | NST | A | 2 | 110 | 0 |
| **8** | 44 | NST | A | 2 | 120 | 0 |
| **9** | 53 | NST | A | 2 | 120 | 1 |
| **11** | 77 | NST | A | 1 | 165 | 0 |
| **12** | 63 | NST | A | 1 | 130 | 0 |
| **13** | 56 | NST | A | 2 | 230 | 1 |
| **14** | 61 | NST | A | 2 | 140 | 0 |
| **15** | 47 | M | A | 2 | 90 | 0 |
| **17** | 60 | M | A | 1 | 225 | 0 |
| **18** | 82 | L | A | 2 | 195 | 1 |
| **19** | 52 | L | A | 2 | 295 | 0 |
| **21** | 40 | MIXED | A | 1 | 270 | 1 |
| **22** | 42 | NST | A | 2 | 200 | 0 |
| **23** | 62 | NST | A | 2 | 175 | 1 |
| **24** | 87 | NST | A | 1 | 290 | 0 |
| **25** | 83 | NST | A | 2 | 85 | 0 |
| **26** | 79 | L | A | 2 | 175 | 0 |
| **27** | 69 | NST | B | 2 | 250 | 0 |
| **28** | 57 | NST | B | 2 | 200 | 0 |
| **29** | 28 | NST | B | 3 | 210 | 0 |
| **31** | 57 | NST | B | 1 | 270 | 1 |
| **32** | 80 | NST | B | 3 | 60 | 0 |
| **33** | 49 | NST | B | 2 | 80 | 1 |
| **34** | 49 | NST | B | 2 | 200 | 0 |
| **36** | 58 | NST | B | 2 | 215 | 0 |
| **37** | 55 | NST | B | 2 | 155 | 0 |
| **38** | 46 | MI | B | 3 | 300 | 1 |
| **39** | 76 | NST | B | 2 | 205 | 0 |
| **40** | 50 | L | B | 2 | 295 | 1 |
| **42** | 51 | M | B | 3 | 145 | 0 |
| **43** | 65 | M | B | 1 | 5 | 0 |
| **44** | 67 | NST | B | 3 | 140 | 1 |
| **45** | 42 | L | B | 2 | 300 | 1 |
| **46** | 57 | NST | B | 2 | 200 | 1 |
| **47** | 45 | NST | B |  | 185 | 1 |
| **48** | 60 | NST | B | 2 | 110 | 1 |
| **49** | 63 | NST | B | 2 | 130 | 0 |
| **50** | 57 | NST | B | 2 | 180 | 1 |
| **52** | 52 | ME | B |  | 180 | 0 |
| **53** | 56 | NST | B | 3 | 135 | 0 |
| **56** | 42 | NST | B | 3 | 170 | 0 |
| **57** | 58 | NST | B | 2 | 255 | 0 |
| **59** | 63 | NST | B | 3 | 240 | 0 |
| **60** | 49 | NST | B | 2 | 200 | 1 |
| **61** | 67 | NST | B | 2 | 255 | 0 |
| **62** | 55 | NST | H | 3 | 255 | 0 |
| **64** | 46 | NST | H | 3 | 280 | 1 |
| **65** | 55 | NST | H | 2 | 200 | 0 |
| **66** | 44 | NST | H | 2 | 105 | 0 |
| **67** |  | NST | H | 2 | 100 | 0 |
| **68** | 46 | NST | H | 3 | 140 | 0 |
| **69** | 75 | NST | H | 3 | 20 | 0 |
| **70** | 60 | NST | H | 3 | 165 | 1 |
| **71** | 56 | NST | H | 3 | 160 | 0 |
| **72** | 59 | NST | H | 2 | 20 | 1 |
| **73** | 58 | NST | H | 3 | 190 | 0 |
| **74** | 79 | NST | H | 3 | 115 | 1 |
| **75** | 62 | NST | H | 3 | 205 | 0 |
| **77** | 40 | MIXED | H | 2 | 130 | 0 |
| **78** | 36 | NST | H | 2 | 100 | 1 |
| **79** | 56 | NST | H | 3 | 215 | 0 |
| **80** | 42 | NST | H | 3 | 155 | 0 |
| **82** | 46 | NST | H | 2 | 110 | 0 |
| **83** | 47 | NST | H | 3 | 240 | 0 |
| **84** | 52 | NST | H | 3 | 225 | 1 |
| **85** | 58 | NST | H | 3 | 235 | 1 |
| **86** | 55 | NST | N | 3 | 210 | 0 |
| **87** | 59 | NST | N | 3 | 130 | 1 |
| **88** | 62 | NST | N | 2 | 185 | 0 |
| **89** | 31 | NST | N | 3 | 100 | 1 |
| **90** | 33 | NST | N | 3 | 260 | 1 |
| **91** | 67 | NST | N | 3 | 145 | 0 |
| **92** | 70 | NST | N | 3 | 175 | 1 |
| **95** | 38 | NST | N | 3 | 90 | 0 |
| **96** | 57 | NST | N | 3 | 160 | 0 |
| **97** | 80 | NST | N | 3 | 210 | 0 |
| **98** | 54 | NST | N | 3 | 100 | 0 |
| **100** | 60 | NST | N | 3 | 295 | 0 |
| **102** | 37 | MP | N | 3 | 140 | 0 |
| **103** | 60 | NST | N | 3 | 280 | 1 |
| **104** | 62 | NST | N | 2 | 225 | 1 |
| **105** | 35 | NST | N | 3 | 115 | 0 |
| **106** | 63 | NST | N | 2 | 225 | 0 |
| **107** | 52 | NST | N | 3 | 150 | 0 |
| **108** | 71 | NST | N | 3 | 280 | 0 |
| **109** | 38 | NST | N | 3 | 170 | 0 |
| **110** | 30 | NST | N | 3 | 230 | 1 |
| **111** | 73 | NST | N | 3 | 125 | 1 |
| **112** | 52 | NST | N | 3 | 230 | 1 |
| **113** | 63 | NST | N | 2 | 270 | 1 |
| **114** | 54 | MI | N | 3 | 210 | 1 |
| **115** | 67 | MI | N | 2 | 155 | 1 |
| **116** | 55 | NST | N | 3 | 275 | 1 |
| **117** | 46 | NST | N | 3 | 265 | 1 |
| **118** | 56 | L | N | 2 | 290 | 1 |
| **119** | 84 | NST | N | 3 | 255 | 1 |
| **121** | 42 | NST | N | 3 | 195 | 1 |
| **122** | 60 | NST | N | 3 | 185 | 1 |
| **123** | 29 | NST | N | 3 | 295 | 1 |
| **124** | 66 | NST | N | 3 | 300 | 1 |
| **125** | 92 | L | N | 2 | 240 | 1 |
| **126** | 60 | MP | N | 3 | 230 | 1 |
| **127** | 49 | NST | N | 3 | 195 | 1 |
| **128** | 38 | NST | N | 3 | 295 | 1 |
| **129** | 62 | NST | N | 3 | 240 | 1 |

**Supplementary references:**

1. Guedj M, Marisa L, de Reynies A, Orsetti B, Schiappa R, Bibeau F, et al. A refined molecular taxonomy of breast cancer. Oncogene [Internet]. 2012 Mar 1;31(9):1196–206. Available from: http://www.ncbi.nlm.nih.gov/pubmed/21785460

2. Maubant S, Tesson B, Maire V, Ye M, Rigaill G, Gentien D, et al. Transcriptome analysis of Wnt3a-treated triple-negative breast cancer cells. PLoS One [Internet]. 2015;10(4):e0122333. Available from: http://www.ncbi.nlm.nih.gov/pubmed/25848952

3. Sabatier R, Finetti P, Cervera N, Lambaudie E, Esterni B, Mamessier E, et al. A gene expression signature identifies two prognostic subgroups of basal breast cancer. Breast Cancer Res Treat [Internet]. 2011 Apr;126(2):407–20. Available from: http://www.ncbi.nlm.nih.gov/pubmed/20490655

4. Kauffmann A, Gentleman R, Huber W. arrayQualityMetrics--a bioconductor package for quality assessment of microarray data. Bioinformatics [Internet]. 2009 Feb 1;25(3):415–6. Available from: http://www.ncbi.nlm.nih.gov/pubmed/19106121

5. Gentleman RC, Carey VJ, Bates DM, Bolstad B, Dettling M, Dudoit S, et al. Bioconductor: open software development for computational biology and bioinformatics. Genome Biol [Internet]. 2004;5(10):R80. Available from: http://www.ncbi.nlm.nih.gov/pubmed/15461798

6. Ritchie ME, Phipson B, Wu D, Hu Y, Law CW, Shi W, et al. limma powers differential expression analyses for RNA-sequencing and microarray studies. Nucleic Acids Res [Internet]. 2015 Apr 20;43(7):e47. Available from: http://www.ncbi.nlm.nih.gov/pubmed/25605792

7. Leek JT, Johnson WE, Parker HS, Jaffe AE, Storey JD. The sva package for removing batch effects and other unwanted variation in high-throughput experiments. Bioinformatics [Internet]. 2012 Mar 15;28(6):882–3. Available from: http://www.ncbi.nlm.nih.gov/pubmed/22257669

8. Marusiak AA, Edwards ZC, Hugo W, Trotter EW, Girotti MR, Stephenson NL, et al. Mixed lineage kinases activate MEK independently of RAF to mediate resistance to RAF inhibitors. Nat Commun [Internet]. 2014 May 22;5:3901. Available from: http://www.ncbi.nlm.nih.gov/pubmed/24849047

9. Subramanian A, Tamayo P, Mootha VK, Mukherjee S, Ebert BL, Gillette MA, et al. Gene set enrichment analysis: a knowledge-based approach for interpreting genome-wide expression profiles. Proc Natl Acad Sci U S A [Internet]. 2005 Oct 25;102(43):15545–50. Available from: http://www.ncbi.nlm.nih.gov/pubmed/16199517

10. Goldhirsch A, Winer EP, Coates AS, Gelber RD, Piccart-Gebhart M, Thürlimann B, et al. Personalizing the treatment of women with early breast cancer: highlights of the St Gallen International Expert Consensus on the Primary Therapy of Early Breast Cancer 2013. Ann Oncol [Internet]. 2013 Sep;24(9):2206–23. Available from: https://academic.oup.com/annonc/article-lookup/doi/10.1093/annonc/mdt303

11. Pirker R, Pereira JR, von Pawel J, Krzakowski M, Ramlau R, Park K, et al. EGFR expression as a predictor of survival for first-line chemotherapy plus cetuximab in patients with advanced non-small-cell lung cancer: analysis of data from the phase 3 FLEX study. Lancet Oncol [Internet]. 2012 Jan;13(1):33–42. Available from: http://linkinghub.elsevier.com/retrieve/pii/S1470204511703187

12. Cancer Genome Atlas Research Network, Weinstein JN, Collisson EA, Mills GB, Shaw KRM, Ozenberger BA, et al. The Cancer Genome Atlas Pan-Cancer analysis project. Nat Genet [Internet]. 2013 Oct;45(10):1113–20. Available from: http://www.ncbi.nlm.nih.gov/pubmed/24071849

13. Burstein MD, Tsimelzon A, Poage GM, Covington KR, Contreras A, Fuqua SAW, et al. Comprehensive genomic analysis identifies novel subtypes and targets of triple-negative breast cancer. Clin Cancer Res [Internet]. 2015 Apr 1;21(7):1688–98. Available from: http://www.ncbi.nlm.nih.gov/pubmed/25208879
